# Hemagglutinin and fusion proteins precede measles virus ribonucleoproteins and marshal cell-to-cell spread in airway epithelium

**DOI:** 10.1128/spectrum.02297-26

**Published:** 2026-10-14

**Authors:** Stephanie E. Clark, Justin W. Kaufman, Kalpana Yadav, Roberto Cattaneo, Brajesh K. Singh, Patrick L. Sinn

**Affiliations:** 1 Microbiology and Immunology, Carver College of Medicine, The University of Iowa 4083, Iowa City, lowa, USA; 2 Stead Family Department of Pediatrics, Carver College of Medicine, The University of Iowa 4083, Iowa City, lowa, USA; 3 Department of Molecular Medicine, Mayo Clinic 4352, Rochester, Minnesota, USA; Emory University School of Medicine, Atlanta, Georgia, USA

**Keywords:** MFA, RNP, glycoproteins, junctions, pseudovirus

## Abstract

Measles virus (MeV) is the most contagious human respiratory pathogen. To understand how it spreads in the airways, we infected well-differentiated primary cultures of human airway epithelial cells (HAE). We discovered that the MeV attachment protein (hemagglutinin, H), the fusion (F) protein, and the assembly organizer protein (matrix, M) reach newly infected cells ahead of, and independently from, the replicative complex proteins. To characterize the mechanisms of MeV spread, we generated a replication-deficient adenoviral vector expressing the MeV M, F, and H proteins in a doxycycline-inducible manner. This viral vector formed infectious centers that preserved epithelial function like those from MeV infection. To further dissect this process, we generated Ad vectors expressing individual MeV proteins, termed Ad-M, Ad-F, and Ad-H. Transduction of HAE with single, double, and triple Ad vector combinations revealed that Ad-F and Ad-H are necessary and sufficient to form MeV-like infectious centers. MeV enters and spreads within airway epithelia via H protein binding to adherens junction protein, nectin-4. To disrupt spread, we introduced the point mutation H^Y543A^, a mutation previously described to inhibit nectin-4 binding, in the Ad-H vector. Co-transduction of Ad-F and Ad-H^Y543A^ did not result in MeV-like infectious center formation. Finally, we assessed whether the MeV-related paramyxovirus Nipah (NiV) attachment and fusion proteins (G and F, respectively) support cell-to-cell spread. Co-transduction of HAE with Ad-NiV-G and Ad-NiV-F did not result in infectious center formation, suggesting that decoupling of the membrane fusion apparatus function from replication is a unique feature of the MeV envelope glycoproteins.

**IMPORTANCE:**

Measles virus is a highly contagious pathogen. We characterized a mechanism by which measles virus spreads rapidly through the cells that line the human airway in an *ex vivo* model. We show that the proteins of the viral membrane fusion apparatus, hemagglutinin (H) and fusion (F), move independently from and reach newly infected cells *before* the proteins of the replicative complex. Thus, viral protein spread is assembled and arranged in order (i.e., *marshaled*). Using adenovirus-based vectors, we show that co-expression of H and F proteins promotes rapid sequential spread to multiple cells and the formation of infectious centers. Further elucidation of the mechanism of measles virus amplification and cell-to-cell spread within the lung may help explain its highly contagious nature.

## INTRODUCTION

Measles virus (MeV) is a negative-sense single-stranded RNA virus belonging to the *Morbillivirus* genus, in the *Paramyxoviridae* family. Related respiratory viruses in this family include Nipah virus (NiV), mumps virus, and parainfluenza viruses. Despite having similar genomic structures with other paramyxoviruses, MeV is the most communicable within unvaccinated populations (1). Indeed, MeV is widely considered the most contagious human virus. The precise mechanism underlying why MeV is more contagious compared to other respiratory viruses is unknown.

**Fig 1:**
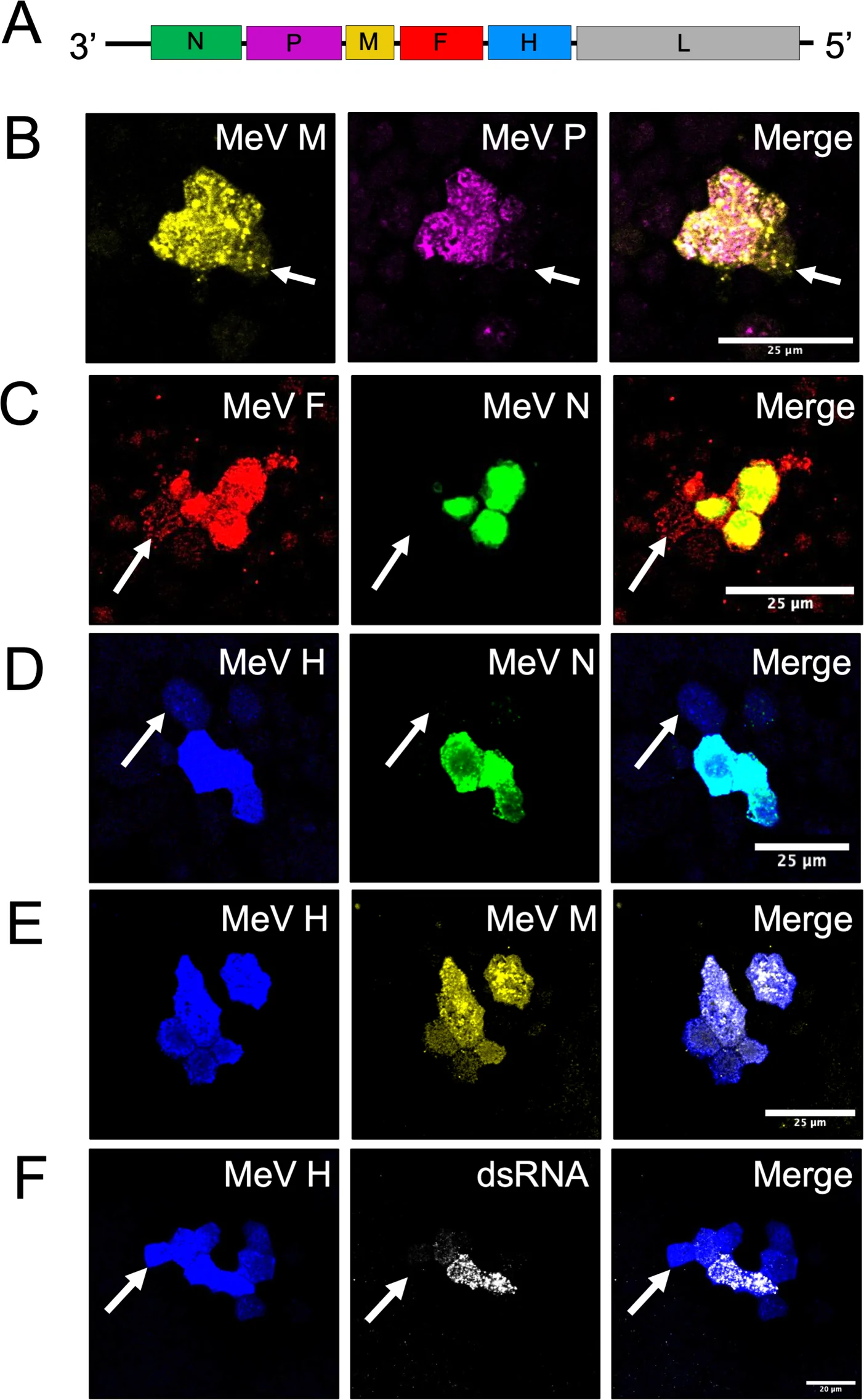
MeV H, F, and M proteins precede N or P in cell-to-cell spread. (**A**) A schematic of the MeV genome is shown, where the protein colors correspond to the pseudocoloring choice of immunocytochemistry fluorescence. (**B–F**) HAE were infected with MeV-nYFP at MOI 1 for 3 h. After 2 dpi, cells were immunostained and imaged via confocal microscopy. Arrows indicate newly infected cells during the process of infectious center formation. The following combination of proteins was visualized: (B) MeV M (yellow) and MeV P (magenta); (**C**) MeV F (red) and MeV N (green); (**D**) MeV H (blue) and MeV N (green); (**E**) MeV H (blue) and MeV M (yellow). Scale bars (**B–E**) = 25 µm. (**F**) MeV H (blue) and dsRNA (white) were co-labeled and imaged. Scale bar = 20 µm.

The MeV genome encodes eight proteins, including six structural proteins (N, P, M, F, H, and L; Fig. 1A) as well as two non-structural proteins (V and C) that function as virulence factors during infection. The MeV ribonucleoprotein (RNP) complex is comprised of nucleoprotein (N) entwined with the RNA genome, in close association with phosphoprotein (P) and the RNA-dependent RNA polymerase (L) (2–4). The RNP complex protects, transcribes, and replicates the genome. A second complex, termed the membrane fusion apparatus (MFA), includes the fusion (F) and hemagglutinin (H) oligomeric glycoproteins and the assembly organizer matrix (M) protein. The MFA complex controls cell entry and cell-to-cell spread (5–10).

To model MeV infection and spread in airway cells, we use well-differentiated primary cultures of human airway epithelial (HAE) cells. These cells are collected from human donor lungs, seeded on permeable polyester support membranes, and grown at an air-liquid interface without passage (11). This pseudostratified cell layer is highly representative of *in vivo* airway epithelia (12, 13). Because these cells are unpassaged, there are a limited number of HAE cultures per donor. MeV infection of HAE results in foci of infection termed “infectious centers” (14). In immortalized cell lines, MeV causes membrane fusion that results in rapid syncytia formation and cell death. In stark contrast, HAE infectious centers form with no cytopathic effect (14–17). MeV-infected HAE within infectious centers retain plasma membranes, individual nuclei remain isolated (5), and transepithelial electrical resistance, which indicates epithelial integrity, remains constant for weeks post-infection (14, 17). Importantly, tissue collected from experimentally infected macaques (18) or infection of freshly isolated human tracheal explants (11) shows that *in vivo* cell-to-cell spread mirrors HAE, confirming model relevance.

MeV infection in airway epithelia is initiated through binding of nectin-4, a cell adhesion protein that serves as the MeV epithelial receptor, located at the basolateral surface (5, 14, 19–22). Subsequently, nectin-4-dependent cell-to-cell spread is facilitated by the formation of pores between an infected cell and an adjacent cell (5). Receptor binding by the H protein head causes a conformational change of its stalk, which triggers refolding of the F trimer and the formation of fusion pores between the plasma membrane of an infected cell and a proximal cell (8, 23–25). Both glycoproteins are required not just for initial infection but also for subsequent cell-to-cell spread (25, 26). While fusion events have been analyzed in immortalized cells, we sought to observe successive lateral spread during MeV infection in primary epithelia during infectious center formation.

After initial entry in HAE, MeV continues to spread rapidly cell-to-cell for 3–5 days, at which point the fully-formed infectious center detaches from the HAE *en masse*. Detached infectious centers contain cell-associated infectious virus (17, 27). Very little free virus is observed both in HAE and immortalized cell models of MeV, suggesting virus remains almost entirely cell-associated during infection. This unique formation of infectious centers in HAE sets MeV apart from other respiratory viruses, as no other human respiratory virus we have screened to date forms infectious centers (5, 19–22).

MeV infection of permissive immortalized cells results in unimpeded fusion pore expansion; however, the circum-apical cytoskeletal rings of HAE may constrain fusion pore expansion, thus preserving epithelial function (28). Using a MeV expressing a fluorescent protein linked to its RNP (termed MeV-RNP^tracker^), we previously reported rapid movement along the circum-apical F-actin rings of newly infected cells, allowing the RNP to reach new cells every 1–2 h (17). We sought to understand how MeV could spread to newly infected cells faster than the time it takes for a single cycle of replication. These observations led us to hypothesize that viral proteins alone may be sufficient to initiate spread. Here, we further characterize how distinct viral protein complexes impact this process by generating an MeV-MFA^tracker^ virus. To determine sufficiency, we also generated adenovirus-based vectors expressing the MeV MFA proteins, alone or in combination. We show here that co-expression of F and H is sufficient to form infectious centers and that these proteins precede the spread of RNP complexes during MeV infection in HAE. This novel mechanism of rapid cellular spread may provide MeV access to an apically inaccessible receptor and permit rapid cellular dissemination in HAE.

## RESULTS

### MFA complex proteins move independently from ribonucleoproteins during cell-to-cell spread

Using a standard infection protocol (15, 17, 29, 30), MeV (wild-type strain IC-323) was applied to the basolateral surface of HAE (multiplicity of infection (MOI) = 1) by inverting the cultures for 4 h. Following infection, the cultures were returned to the original upright position. To visualize infected cells, MeV expressing a nuclear-localized yellow fluorescent protein (MeV-nYFP) was utilized. At 2 days post-infection (dpi), viral protein localization was visualized by immunocytochemistry and confocal microscopy. This timepoint represents an early stage of HAE infection when lateral cell-to-cell spread is just beginning from the initially infected cell. Imaging detected the MeV M, F, and H proteins before the MeV N and P proteins in laterally infected cells (Fig. 1B and C and D). N protein is the first protein produced in the viral genome and highest in abundance; thus, protein abundance does not dictate the order in which they are detected in newly infected cells. H protein and M protein co-occupy all infected cells (Fig. 1E). We next sought to determine if cell-to-cell spread precedes viral replication. To do this, cells were immunostained for MeV H and dsRNA, which can be detected at sites of MeV genome replication (Fig. 1F). We observed dsRNA staining most robustly in the earliest infected cells, with little to no signal observed in laterally located infected cells alongside MeV H. These results suggest that MFA proteins associated with spread (i.e., M, F, and H) move independently from and precede RNP proteins associated with replication (i.e., N and P).

**Fig 2:**
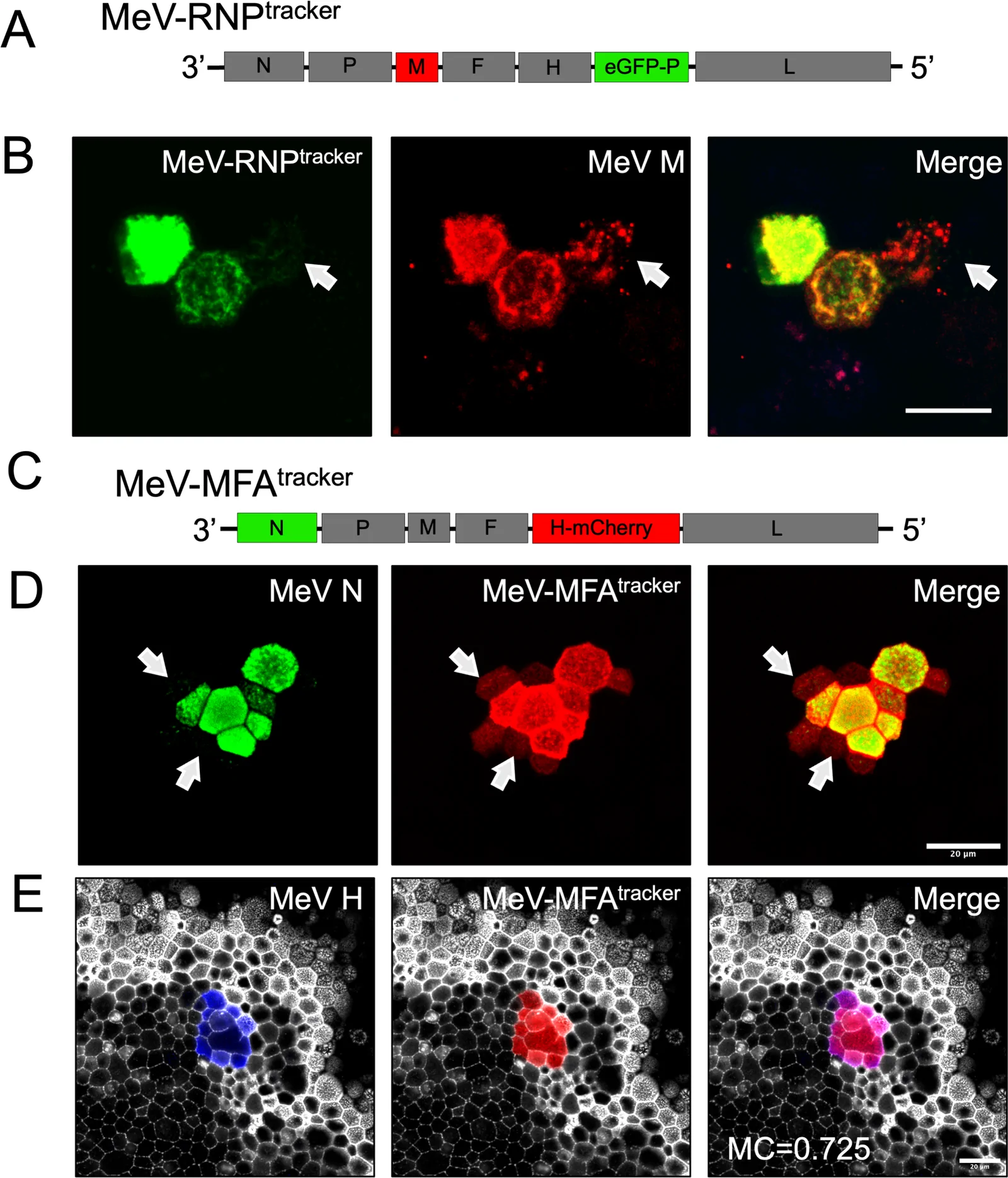
Cell-to-cell spread of MFA^tracker^ and RNP^tracker^ measles viruses**.** (**A**) A schematic of MeV-RNP^tracker^ with eGFP-tagged P is shown. (**B**) HAE were infected with MeV-RNP^tracker^ at MOI 1 for 4 h and imaged at 2 dpi. Immunocytochemistry was performed for MeV M protein (red), and co-localization with RNP^tracker^ was imaged via confocal microscopy. Scale bar = 20 µm. (**C**) A schematic of MeV-MFA^tracker^ with mCherry-tagged H is shown. (**D**) HAE were infected with MeV-MFA^tracker^ at MOI 1 for 4 h and collected at 2 dpi. Immunocytochemistry was performed for MeV N protein (green) and co-localization with MFA^tracker^ was imaged via confocal microscopy. Scale bar = 20 µm. (**E**) HAE were infected with MeV-MFA^tracker^ at MOI 1 for 4 h and collected 2 dpi. Immunocytochemistry was performed for MeV H protein (blue) and F-actin phalloidin (white), and co-localization with MFA^tracker^ was imaged via confocal microscopy. Scale bar = 20 µm. Manders coefficient (MC) was calculated utilizing JACoP plugin in Fiji.

To analyze the kinetics of cell-to-cell spread of the MeV complexes, viruses allowing direct visualization of the RNP (MeV-RNP^tracker^) (28) or of the MFA (MeV-MFA^tracker^) were utilized. As shown schematically in Fig. 2A, MeV-RNP^tracker^ expresses a second P protein tagged with eGFP. For live-cell imaging, HAE were infected, and imaging began at 24 hpi. Movie S1 shows that RNP fluorescence was primarily punctate when it appeared in newly infected cells. These observed RNP puncta may be sites of transcription and replication occurring simultaneously with cell-to-cell spread and infectious center formation. Complementary immunohistochemistry analysis of MeV-RNP^tracker^ -infected HAE 2 dpi detected M protein before tagged P (Fig. 2B), consistent with observations of Fig. 1B.

To observe live-cell movement of the MFA, a construct with a mCherry-tagged H protein was generated (MeV-MFA^tracker^; Fig. 2C). HAE were infected, and imaging began at 24 hpi and continued over the course of 72 h. H-tagged protein signal was diffuse, in contrast to the puncta signal of the P-tagged protein (Movie S2). This further suggests that movement of MFA proteins in the cell occurs independently from the punctate localization of RNP proteins, potentially due to each complex performing different functions during critical cell-to-cell spread events early in infection. At 2 dpi, N-protein and tagged H-protein were visualized by immunocytochemistry. Only tagged H-protein was visualized in outermost infected cells (Fig. 2D), consistent with the observations of Fig. 1D. A control experiment verified that the MeV H and mCherry-tag signals co-localize (Fig. 2E). Co-localization of MeV P and GFP-tag of MeV-RNP^tracker^ was previously described (28). These analyses suggest that the MFA proteins move independently from and reach newly infected cells before the replicative complex proteins.

### MFA complex proteins precede ribonucleoproteins in newly infected cells

**Fig 3:**
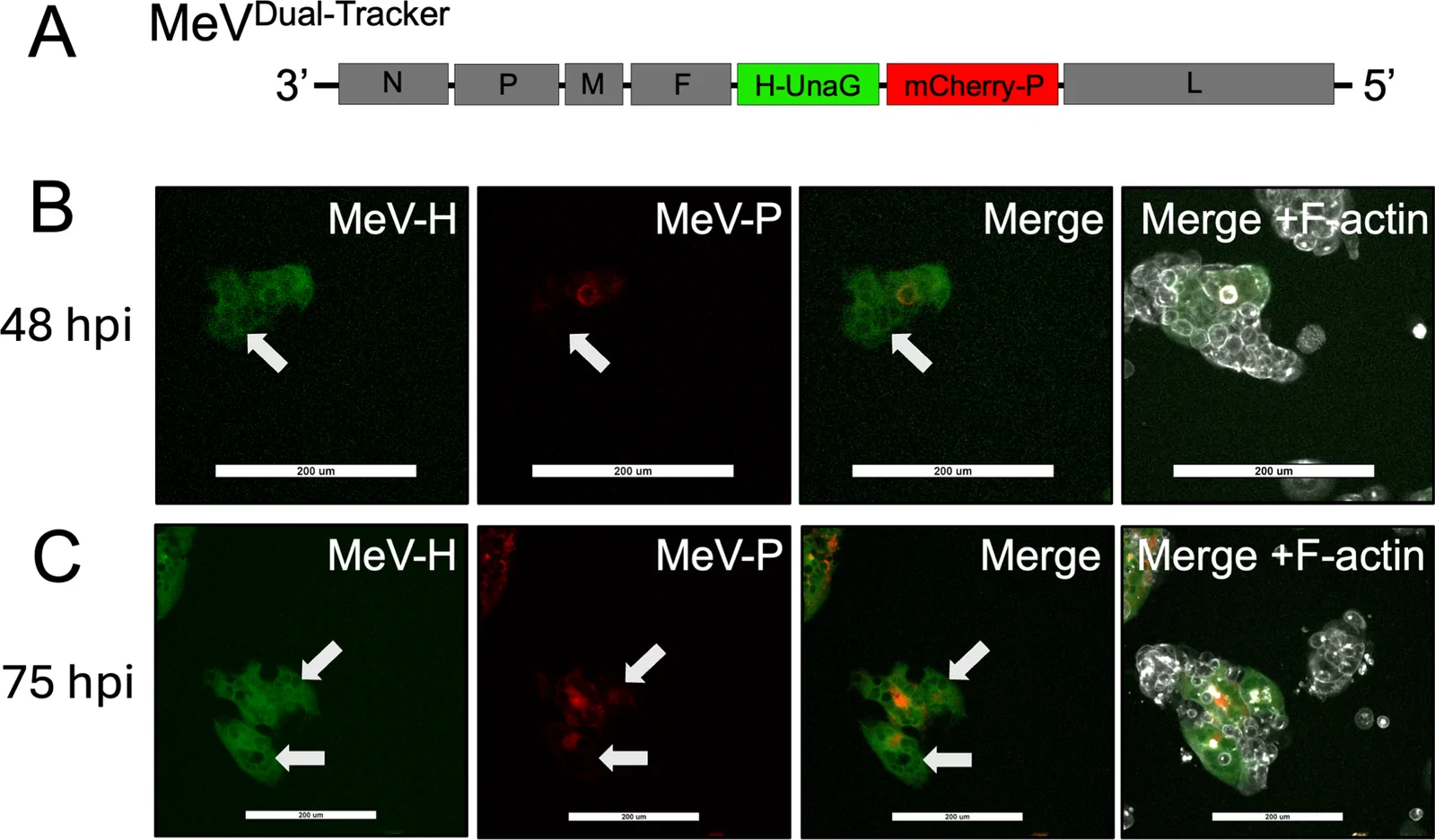
Live cell imaging of a dual-tracker MeV**.** (**A**) A schematic of MeV^Dual-Tracker^ with UnaG-tagged H and mCherry-tagged P is shown. H358 cells were infected with MeV^Dual-Tracker^ at MOI 0.1 for 2 h. At 23 hpi, cells were incubated in media with 1 μM SiR-Actin 647 dye (white) and 10 μM verapamil 1 h before the start of live imaging. Verapamil is an efflux pump inhibitor that reduces cellular expulsion of the dye and prolongs imaging time. The time-lapse movie was started at 24 hpi, and SiR-Actin 647 and verapamil remained in media throughout imaging. Images were collected every 0.5 h for 72 h. (**B and C**) Two different frames from the Movie S3 at 48 and 75 hpi are shown. Arrows indicate regions that are H-positive and P-negative. During imaging, cells were maintained at 37°C, 5% CO_2_. Scale bars = 200 µm.

To directly compare the kinetics of MFA and RNP cell-to-cell spread, a “dual tracker” virus was generated where both an MFA and an RNP protein are fluorescently tagged. MeV^Dual-Tracker^ expresses a second P protein tagged with mCherry and an H protein tagged with UnaG (Fig. 3A). For logistical reasons that limit UnaG detection in cells grown on transwell filters, we used H358 cells grown on cell culture plates. H358 cells were infected with MeV^Dual-Tracker^ at an MOI of 0.1 for 2 h in 24-well glass-bottom plates. To visualize the cytoskeleton of individual cells during analysis, a live F-actin dye, SiR-actin, was utilized. Starting at 24 hpi, cells were imaged every 30 min for 72 h (Movie S3). Patterns of RNP and MFA spread in H358 cells were consistent with spread in HAE. In newly infected cells, tagged-MeV H appears first and fills the cell body, while the punctate P protein signal appears in new cells later in infection (Fig. 3D; Movie S3). Thus, the MeV MFA complex precedes the RNP complex during cell-to-cell spread in both immortalized and primary airway epithelial cells.

### Inhibition of viral RNA polymerase limits MeV cell-to-cell spread

**Fig 4:**
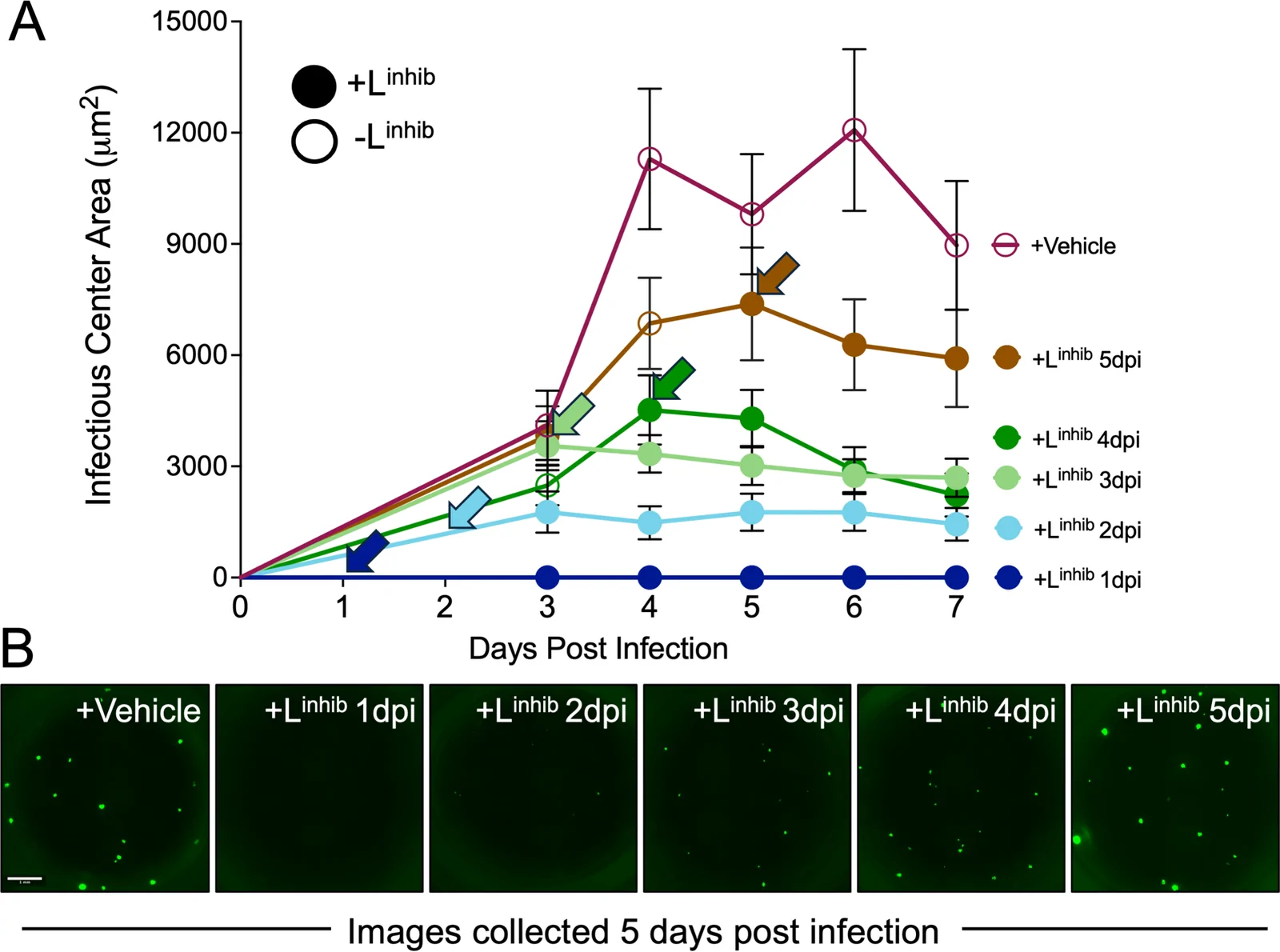
Inhibition of viral RNA polymerase limits MeV cell-to-cell spread. (**A**) HAE were infected with MeV-GFP at MOI 0.5 for 3 h. Media was refreshed daily. At progressing time points post-infection (1–5 dpi), DMSO (vehicle) or 50 µM L^inhib^ (ERDRP-0159) was added to the basolateral media. HAE were imaged daily from 3 to 7 dpi via fluorescent microscopy. The areas of infectious centers were quantified with Fiji. Open circles indicate vehicle treatment, and closed circles indicate 50 µM L^inhib^ treatment. Arrows with corresponding colors to each dpi indicate the beginning time point at which L^inhib^ was added. (**B**) Representative images are shown at 5 dpi. Scale bar = 1 mm. *n* = all infectious centers measured per time point and combined from two donors (Table S1).

We next asked if an established MeV infection will continue to spread cell-to-cell when viral replication is inhibited. We hypothesized that once MFA levels reach a critical level in an initially infected cell, cell-to-cell spread will initiate and continue into more cells in the absence of new viral protein or genome replication. To inhibit MeV RNA-dependent RNA polymerase, we used non-nucleoside inhibitor ERDRP-0159 (termed here L^inhib^) (31–34). We infected HAE with MeV-eGFP using the standard protocol. At successive timepoints post-infection, 50 μM L^inhib^ or DMSO (mock) was added to the basolateral media from 1 to 5 dpi (Fig. 4A). HAE were imaged daily from 3 to 7 dpi on a Keyence microscope (Fig. 4B) and analyzed in Fiji (Table S1). Infectious center size was measured as an indicator of cell-cell spread (17, 30). At 1 dpi, inhibitor addition prevented infectious center formation. At 2–5 dpi, addition of L^inhib^ resulted in cessation or reduction of infectious center growth. Contrary to our hypothesis, we did not observe continued spread without replication. This suggests that continuation of lateral cell-to-cell spread may require a constant replenishment of an MFA protein gradient.

### The MFA proteins can drive cell-to-cell spread alone

**Fig 5:**
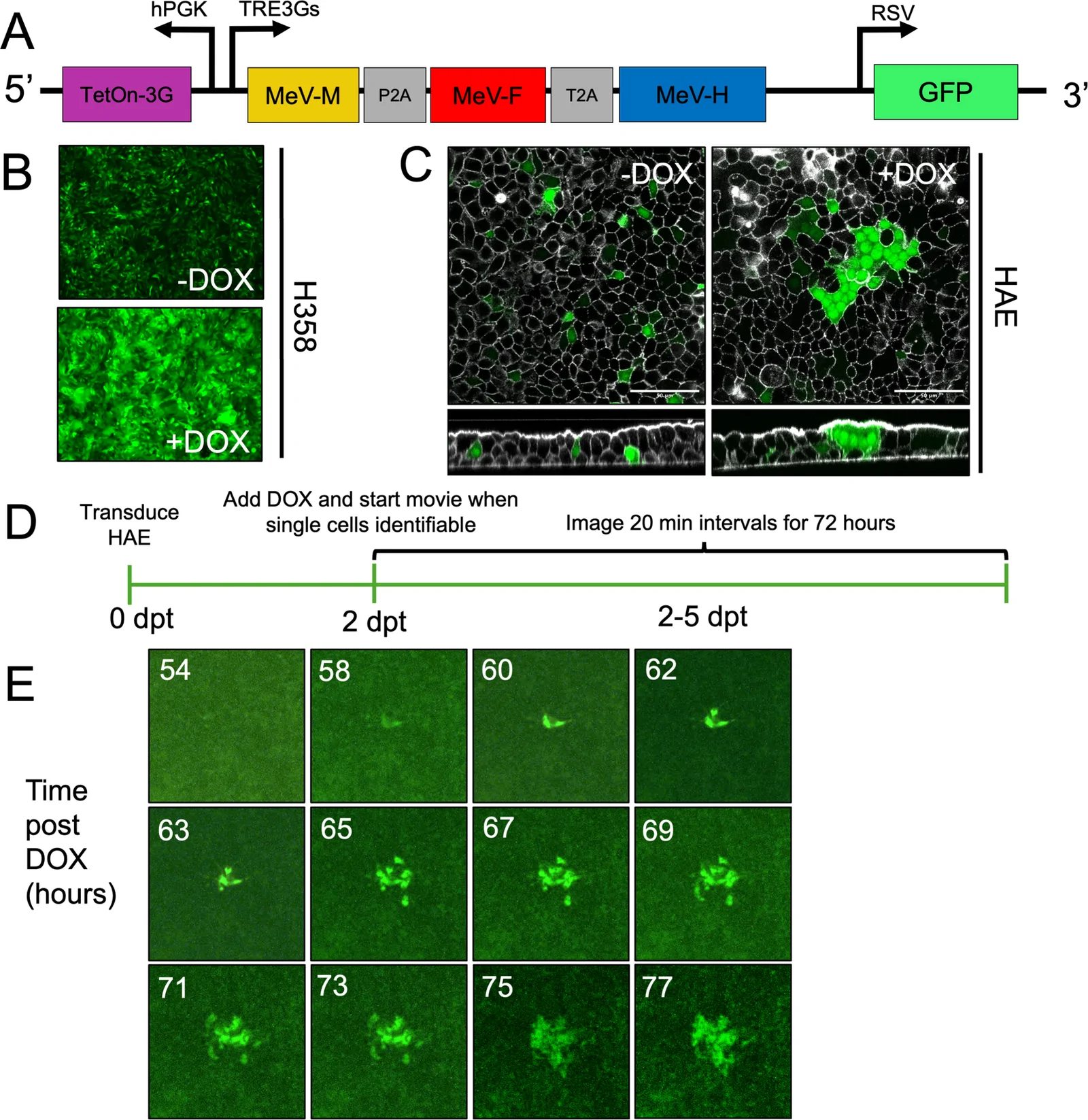
Ad-MFH facilitates cell-to-cell spread and recapitulates WT MeV. (**A**) A schematic of Ad-MFH vector design is shown. The vector includes M, F, and H, separated by P2A and T2A elements and driven by a tetracycline-inducible promoter. A downstream constitutively active eGFP reporter gene is driven by a Rous sarcoma virus (RSV) promoter. (**B**) H358 cells were transduced with Ad-MFH at an MOI of 50 for 2 h. Doxycycline (1 µg/mL; +DOX) or DMSO (−DOX) was added and imaged 2 days post-transduction (dpt) via fluorescent microscopy. (**C**) HAE were transduced with Ad-MFH at MOI 100 for 2 h applied to the apical surface. Doxycycline (1 µg/mL; +DOX) or DMSO (−DOX) was added in the basolateral media. Both conditions were fixed, counterstained with F-actin phalloidin (white), and imaged 5 dpt via confocal microscopy. Scale bar = 50 µm. (D) A timeline of the Ad-MFH transduction and time-lapse movie is shown. Briefly, HAE were transduced with Ad-MFH at an MOI of 50 for 2 h applied to the apical surface. After singly transduced cells were visible by eGFP fluorescence at 2 dpt, 1 µg/mL doxycycline was added to the basolateral medium to start the expression of MeV proteins. Images were collected every 20 min for 72 h. During imaging, cells were maintained at 37°C, 5% CO_2_. (**E**) Selected timepoints from the Ad-MFH time-lapse movie from 54 to 78 h post-DOX addition are shown.

To assess whether the MFA proteins can drive cell-to-cell spread in the absence of the MeV RNP proteins, a helper-dependent adenovirus (Ad) vector was generated expressing MeV M, F, and H proteins separated by picornavirus 2A elements (termed Ad-MFH; Fig. 5A) (35). A tetracycline-responsive (TetON) promoter activates polycistronic gene expression in the presence of doxycycline (DOX), as confirmed by immunoblot (Fig. S1). A constitutively expressed eGFP reporter gene was included to identify transduced cells. We use “infect” to indicate delivery of a replication-competent virus, whereas “transduce” indicates delivery of a replication-incompetent viral vector. Ad vectors were chosen because fully differentiated HAE are resistant to transfection; however, HAE are readily transduced with Ad vectors (36). Transduction of H358 cells with Ad-MFH results in individual GFP+ cells in the absence of DOX. Syncytia form following application of DOX into the media (Fig. 5B). Of note, transduction of HAE with Ad-MFH results in individual GFP+ cells in the absence of DOX, but in the presence of DOX, clusters of GFP+ cells that resemble infectious centers were observed (Fig. 5C). Here, we term these clusters pseudo-infectious centers (Fig. S2A). Live cell imaging (Fig. 5D) of Ad-MFH revealed cell-cell spread via cytoplasmic sharing (Fig. 5E and Movie S4), similar to bona fide MeV infection (Fig. S2B and Movie S5). These results indicate that the MFA proteins can drive cell-to-cell spread in the absence of the RNP proteins.

### Ad-MFH induces epithelial-to-mesenchymal transition in HAE

A striking phenotype of infectious center formation in HAE is the loss of junctional markers between cells. We previously reported that MeV infection leads to activation of epithelial-to-mesenchymal transition (EMT), a process in which cells transition from a more epithelial-like to more mesenchymal-like phenotype (29). This transition is marked by loss of junctional protein markers and structured actin in circum-apical rings. Examples of junctional protein markers include F-actin, ZO-1, and E-cadherin. Similar to infectious centers, formation of pseudo-infectious centers resulted in loss of junctional markers (Fig. S2A). Loss of junctional markers was not observed with Ad5-GFP vector transduction alone in the absence of MeV proteins (Fig. S3B). These data confirm that expression of the MeV MFA proteins, without expression of the RNP proteins or viral replication, is sufficient to induce changes to the HAE that facilitate spread.

### Only MeV F and H are required for the formation of pseudo-infectious centers

**Fig 6:**
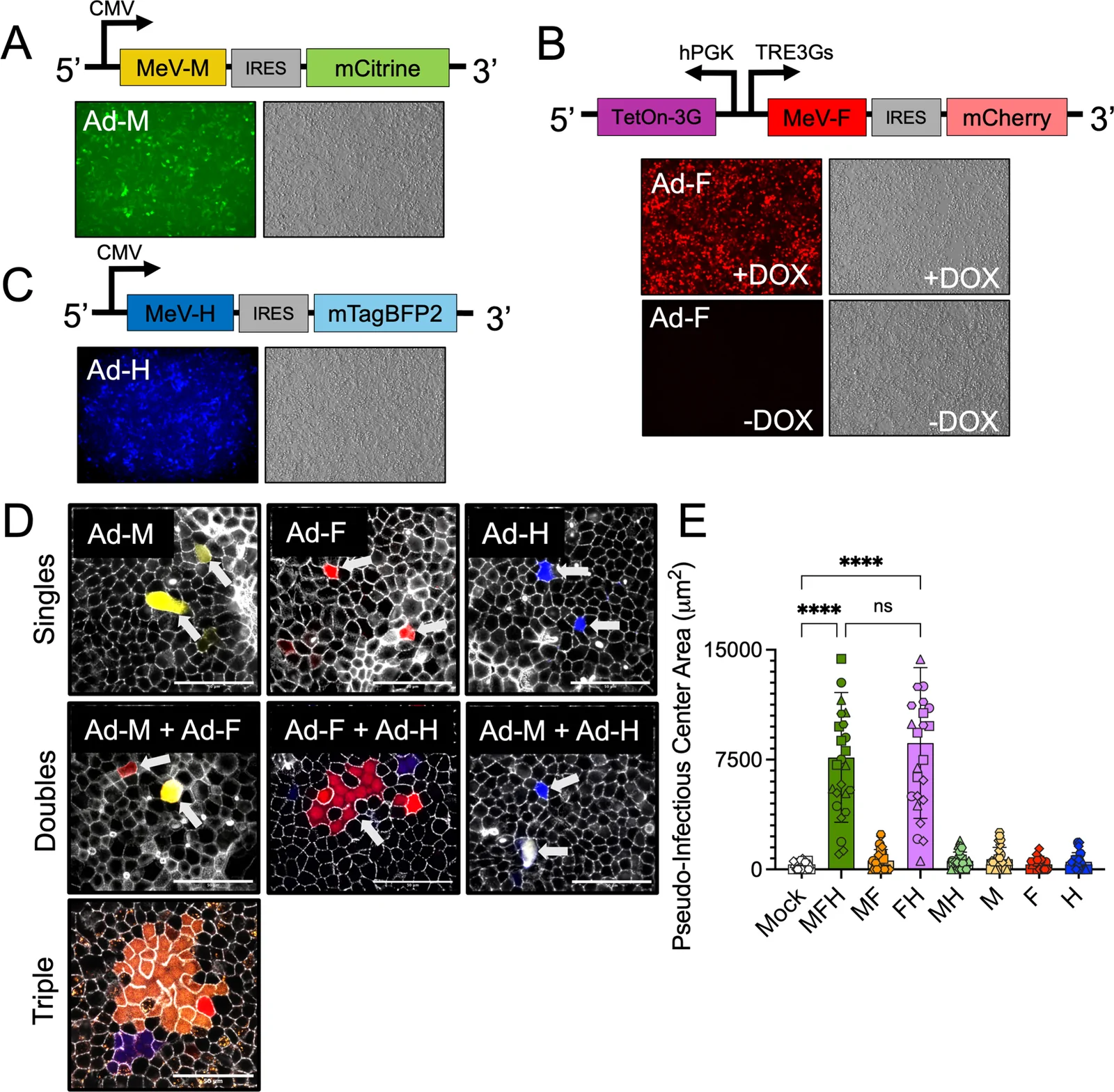
MeV H and F are minimally necessary for cytoplasmic sharing events. (**A**) As indicated schematically, Ad-M includes bicistronic mCitrine reporter gene expression. Ad-M transduced H358 cells (MOI 50) were imaged for fluorescence and brightfield via Keyence. (**B**) Ad-F includes bicistronic mCherry reporter gene expression and is driven by a tetracycline-inducible promoter. Ad-F transduced H358 cells (MOI 50) were imaged for fluorescence and brightfield via Keyence. After transduction, 1 µg/mL doxycycline was added to induce expression of MeV-F and mCherry fluorophore. (**C**) Ad-H includes bicistronic mTagBFP2 reporter gene expression. Ad-H transduced H358 cells (MOI 50) were imaged for fluorescence and brightfield via Keyence. (**D**) Single, double, and triple vector combinations of Ad-M, Ad-F, and Ad-H were applied to the apical surface of HAE for 2 h. Mock conditions were donor matched. After 5 dpt, cells were fixed and counterstained with F-actin phalloidin (white). Scale bar = 50 µm. (**E**) Loss of F-actin was measured via blinded 40× images of each HAE culture of the phalloidin staining alone (without vector fluorescence) and analyzed in Fiji. *n* = 5 donors, each shape is a different donor, and each point is a different HAE culture. ns, not significant; *P < 0.05 and ****P < 0.0001; mixed-effects analysis with multiple comparisons.

To further analyze the function of the three MeV MFA proteins, we generated adenoviral vectors that express these proteins individually. These vectors, termed Ad-M, Ad-F, and Ad-H, also express the fluorescent proteins mCitrine, mCherry, or mTagBFP2, respectively (Fig. 6A through C). Each vector was tested in H358 cells (MOI 50) to confirm fluorescence (Fig. 6A through C) and protein expression by immunoblot (Fig. S1A through C). HAE were transduced with single, double, and triple combinations (MOI 250) to determine necessity and sufficiency of M, F, and H to facilitate cell-to-cell spread. Single vector transduction, as well as co-transduction with Ad-M/Ad-H or Ad-M/Ad-F, failed to result in cell-to-cell spread, as observed by confocal microscopy (Fig. 6D). Importantly, co-transduction of Ad-F and Ad-H (in the presence or absence of Ad-M) resulted in pseudo-infectious center formation (Fig. 6D and Fig. S2C). As described in Materials and Methods, we used an F-actin-based blinded quantification system to measure pseudo-infectious center area (Fig. 6E). These data indicate that constitutive co-expression of the MeV envelope glycoproteins is sufficient to induce the formation of pseudo-infectious centers in both primary and immortalized airway epithelial.

### Pseudo-infectious center formation is nectin-4 dependent

**Fig 7:**
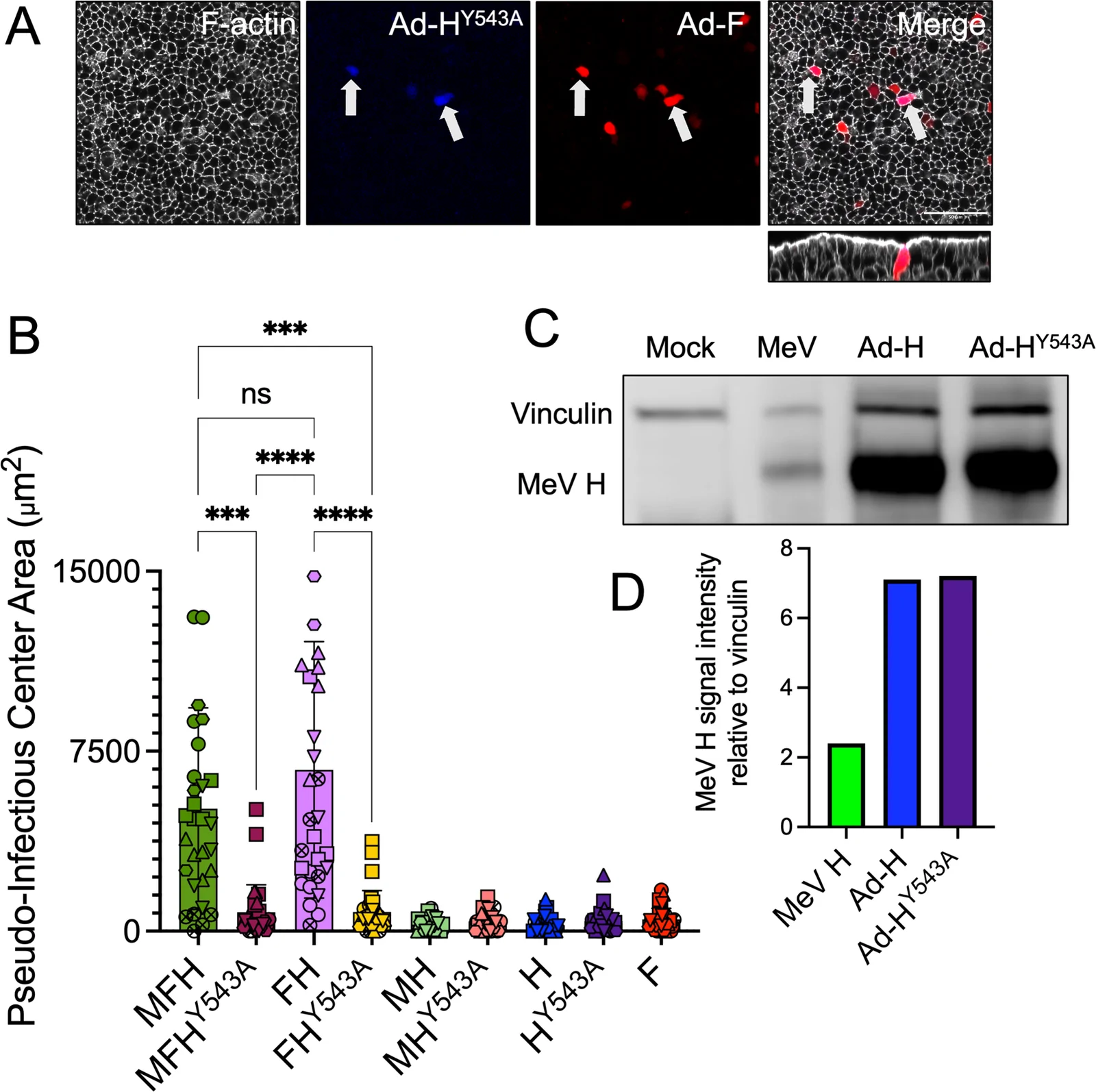
Cytoplasmic sharing events are Nectin-4 dependent. (**A**) HAE were singly or co-transduced with Ad-F (MOI 50) and Ad-H^Y543A^ (MOI 250), for 2 h. After transduction, 1 µg/mL doxycycline was added in the media for the +DOX condition to induce expression of MeV F and mCherry. At 5 dpt, cells were fixed, counterstained with phalloidin (white), and imaged with confocal microscopy. Scale bar = 50 µm. Arrows indicate cells that have been co-transduced by both vectors. (**B**) Loss of F-actin phalloidin was measured in µm^2^ via five blinded, representative 40× images of each HAE culture of the phalloidin staining alone (without vector fluorescence) and analyzed in Fiji. *n* = 6 donors, each shape is a different donor, and each point is a different HAE culture. (**C**) Immunoblot of H358 cells transduced with Ad-H or Ad-H^Y543A^ or infected with MeV-GFP. Cells were immunoblotted for vinculin and MeV-H. (**D**) Immunoblot signal intensity of MeV H over vinculin was measured via LI-COR imager analysis. ns, not significant; ***P < 0.001 and ****P < 0.0001; mixed-effects analysis with multiple comparisons.

Expression of MeV F protein alone has been shown to be sufficient for receptor-independent syncytia formation in immortalized cell lines (Fig. S4) (37, 38). To determine if cell-to-cell spread of vector-expressed glycoproteins in HAE was receptor dependent, we introduced a point mutation in our Ad-H vector at position 543 in the hemagglutinin sequence, changing a tyrosine to an alanine (Ad-H^Y543A^). This amino acid substitution was previously characterized to make MeV H “blind” to nectin-4 but retains the ability to bind to its lymphocytic receptor, SLAMF1 (15). Co-transduction of Ad-H^Y543A^ and Ad-F in HAE failed to confer cell-to-cell spread as observed by confocal microscopy (Fig. 7A) and pseudo-infectious center area via F-actin-based quantification (Fig. 7B). Protein expression levels between Ad-H^Y543A^ and Ad-H were equivalent as determined by immunoblot (Fig. 7C and D). These results indicate that pseudo-infectious center formation in HAE is receptor dependent.

### Nipah virus glycoproteins do not elicit pseudo-infectious centers formation

**Fig 8:**
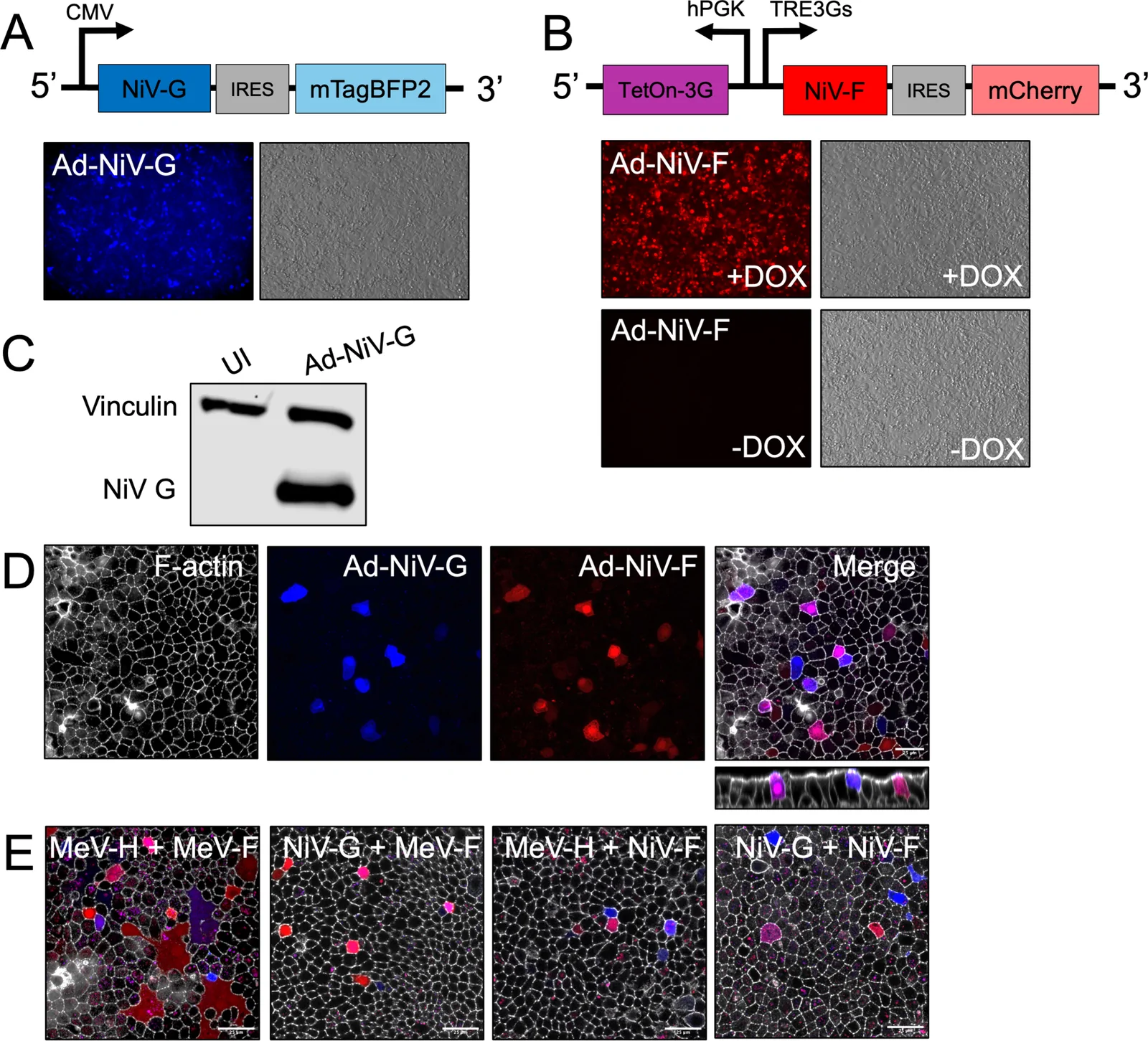
Co-expression of Nipah virus glycoproteins, F and G, does not result in cytoplasmic sharing. (A) As indicated schematically, Ad-NiV-G includes bicistronic mTagBFP2 reporter gene expression. Ad-NiV-G transduced H358 cells (MOI 50) were imaged for fluorescence and brightfield via Keyence. (**B**) Ad-NiV-F includes bicistronic mCherry reporter gene expression and is driven by a tetracycline-inducible promoter. Ad-NiV-F transduced H358 cells (MOI 50) were imaged for fluorescence and brightfield via Keyence. After transduction, 1 µg/mL doxycycline was added to induce expression of NiV-F and mCherry fluorophore. ±DOX conditions were imaged. (**C**) Immunoblotting confirmed NiV protein expression of vinculin and NiV-G in H358 cells. (**D and E**) HAE were co-transduced with (**D**) Ad-NiV-G and Ad-NiV-F or (**E**) combinations of Ad-NiV-G, Ad-NiV-F, Ad-MeV-H, and/or Ad-MeV-F at MOI 250 (for receptor attachment protein) or MOI 50 (for fusion protein) for 2 h. At 5 dpt, cells were fixed, counterstained with phalloidin (white), and imaged with confocal microscopy. Scale bar = 25 µm.

Since NiV, a member of the *Paramyxoviridae* family that uses junctional protein ephrin-B2 as a receptor, spreads laterally in porcine airway epithelial cultures (39), we assessed whether expression of its glycoproteins, fusion (F) and glycoprotein (G), results in formation of pseudo-infectious centers in HAE. Ad vectors expressing the Bangladesh strain of Nipah Fusion (Ad-NiV-F) and Nipah Glycoprotein (Ad-NiV-G; Fig. 8A and B) were codon-optimized and generated in the same vector backbones as MeV Ad-F and Ad-H. Vectors were validated in H358 cells to confirm fluorescence and protein expression by immunoblot and fusion capacity by confocal microscopy (Fig. 8A through C and Fig. S4). Ephrin-B2 expression in HAE was confirmed via a previously deposited single-cell RNA sequencing data set (GSE185048) and immunoblotting (Fig. S5A and B).

We did not detect cell-to-cell spread events in HAE co-transduced with Ad-NiV-F and Ad-NiV-G vectors (Fig. 8D). Co-transduction of different combinations of NiV and MeV glycoproteins did not elicit the formation of pseudo-infectious centers, except for the MeV Ad-F/Ad-H control (Fig. 8E). These data indicate that expression of NiV G and F, alone or in combination with MeV H and F, did not recapitulate the cell-to-cell spread phenotype observed during MeV infection.

## DISCUSSION

In this study, using unpassaged, well-differentiated primary human airway epithelial cells, we demonstrate that early during infectious center formation, MeV proteins organize and propagate through two distinct protein complexes: MFAs and RNPs. MFAs initiate cell-to-cell connectivity, effectively “trailblazing” intercellular pathways, while RNPs follow, facilitating viral replication in the wake of these connections. We observed, at early time points of infection, MFA proteins precede RNP proteins. This observation occurs shortly after infection, and by later time points (4–7 dpi), the two complexes co-occupy the same cells as the infectious centers stop growing and detach. Using replication-incompetent Ad vectors expressing MeV MFA proteins, we recapitulated receptor-dependent cell-to-cell spread, confirming that MeV F and H proteins in combination are both necessary and sufficient to initiate cytoplasmic sharing events. We propose that the independent activity of these viral components during early infectious center formation may confer an evolutionary advantage, enabling rapid dissemination within the airway epithelium.

Cell-to-cell spread offers several advantages over cell-free transmission, including reduced extracellular exposure and evasion of antibody neutralization (40). Importantly, MeV exploits nectin-4, an adherens junction protein, as its receptor (19, 21). Lateral spread between adjacent cells provides access of MeV H to nectin-4. Given that nectin-4 is not apically accessible in HAE (11), apical budding and reinfection of neighboring cells at the surface would be highly inefficient. Previous studies have shown that MeV remains predominantly cell associated in both immortalized and primary cell cultures, including within infectious centers (9, 17, 41), supporting the hypothesis that MeV replication and lateral spread occur primarily through intracellular mechanisms. We sought to understand what unique characteristic about MeV allows for cell-to-cell spread and infectious center formation. Given that the MeV receptor is a junctional protein, we utilized NiV to ask the question: is MeV able to spread cell-to-cell simply because of the presence of a junctional receptor? The epithelial receptor for NiV, ephrin-B2, is also an adherens junction protein (42). However, our data demonstrate that Ad-vector expression of NiV glycoproteins, G and F, alone or in conjunction with MeV glycoproteins, did not recapitulate the cell-to-cell spread phenotype observed during MeV infection. This suggests the possibility of an additional mechanism at play beyond simply localization of viral receptors.

Beyond immune evasion and efficiency, cell-to-cell spread may also leverage cytoskeletal networks to facilitate rapid transmission between infected and uninfected cells. Cytoskeletal structures may serve as hubs for viral protein assembly and trafficking. We previously observed that RNP complexes travel along circum-apical F-actin rings in HAE, and cytoskeletal proteins such as F-actin, E-cadherin, and ZO-1 are progressively lost during infection, likely due to MeV-induced EMT (28, 29). EMT is initiated by diverse stimuli to dissolve cell-cell connections and promote mobilization. EMT has been studied for its involvement in wound repair, tumor metastasis, and response to viral infection, while live cell extrusion (LCE) activates during cell overcrowding, damage repair, and apoptotic signaling (43–48). We recently published that EMT as well as LCE are both activated during MeV infection and contribute to infectious center detachment (29). Interestingly, the pseudo-infectious centers generated by our Ad-expression system appear to undergo EMT (Fig. S3) but do not undergo LCE. During EMT, in addition to loss of cell-cell adhesion, cells gain actomyosin contractility, leading to cell extrusion (44). Indeed, many signaling pathways between EMT and LCE overlap, and the processes are difficult to separate. Pseudo-infectious center formation may provide an opportunity to study the triggers of EMT-mediated LCE. The precise triggers of cytoskeletal breakdown within infectious centers remain unclear, and it is yet to be determined whether this process benefits viral spread or represents a host response to infection.

Another open question arising from this work concerns the role of MeV matrix (M) protein in cell-to-cell spread within HAE. Previous studies suggest that M, in conjunction with RNP, contributes to F-actin remodeling and preferentially co-localizes with actin filaments to facilitate viral spread (49). M may function to localize MFA and RNP complexes during virion assembly (6, 10, 49, 50). We postulate that M assists in MFA formation by spatially coordinating F and H proteins but is not essential for infectious center formation. The role of M in pseudo-infectious center formation may be masked by the super-physiological levels of F and H during Ad-vector expression. Furthermore, Ad-expressed MeV proteins are unlikely to accurately reflect the stoichiometry of a WT MeV infection.

Previous studies have utilized replication-deficient Ad expressing MeV H and/or F as potential live-virus vaccine candidates (51–53). In addition to Ad, VSV and lentiviral vectors expressing MeV F and H have been employed previously not only to study MeV biology but also for therapeutic potential in cancer treatments (54–56). Here, we utilize Ad due to ease of production, high titer generation, and permissibility in HAE. In addition, when a larger cassette size was required for the desired transgene insertion, helper-dependent adenovirus was employed (57). Modular expression of MeV proteins allows for numerous advantages including: (i) the benefit of controlling the start of protein expression via an inducible promoter, (ii) quicker generation of mutants with or without fluorescent markers, and (iii) the avoidance of the need to generate potentially non-viable recombinant MeV mutants. These vectors allow us to define the role of each protein in the MFA alone and isolate protein function away from the confounding impact of genome replication.

It is well-established that MeV utilizes both F and H proteins to mediate fusion in immortalized cell lines (25, 26, 58). In some contexts, overexpression of MeV F alone can induce fusion events independent of H (37, 38). Our findings in HAE show that F and H proteins are required to mediate pore formation and cell-to-cell spread without the involvement of other viral proteins. Of note, we used codon-optimized MeV F and H sequences in Ad vectors. Initial vector iterations failed to express MeV proteins in HAE, likely due to cryptic splice sites that hindered RNA processing in the nucleus, a step not required for cytoplasmic replication. Codon optimization and constitutive heterologous promoters enabled robust protein expression in both Ad and helper-dependent Ad vectors. As a result, Ad conferred protein expression likely exceeds native MeV protein levels, as suggested by the immunoblot in Fig. 7C and Fig. S1.

There are currently no FDA-approved inhibitors to treat MeV infection. In our studies with HAE, utilizing MeV RDRP non-nucleoside inhibitor, ERDRP-0159, we observed that addition of the drug early in infection stagnated infectious center growth and inhibited detachment. If added early enough (1 dpi), infectious center formation was entirely prevented. At later timepoints (4–5 dpi), addition of inhibitor reduced infectious center growth, but detachment still occurred. If we consider infectious center detachment as a correlate of MeV spread, this suggests that this inhibitor could potentially provide not just a therapeutic benefit but also reduce host-to-host transmission in a hospital, daycare, or school setting. Indeed, *in vivo* studies utilizing ERDRP-0159 with large animal models suggest that post-exposure treatment has high potential for clinical success, including reducing symptomatic disease (32, 34).

In summary, this work reveals a two-step mechanism of MeV infectious center formation in HAE, in which MFA complexes initiate cytoplasmic sharing, followed by RNP-mediated genome replication. Using Ad-based vectors, we demonstrate that MeV F and H proteins are both necessary and sufficient to drive receptor-dependent cell-to-cell spread. Our findings suggest that segregation of viral protein complexes enables efficient lateral propagation within airway epithelial cells. This strategy may confer an evolutionary advantage in a tissue where apical reinfection is constrained. Together, these observations refine our understanding of MeV spread in airway cells and may contribute to an explanation for why MeV is more contagious than other respiratory pathogens.

## MATERIALS AND METHODS

### Immortalized cells

Vero-hSLAMF1 cells (59) (Vero cells [ATCC # CCL-81] with stable expression of human Signaling Lymphocytic Activation Molecule Family Member 1 [SLAMF1]) were maintained at 37°C and 5% CO_2_, in Dulbecco’s high-glucose modified Eagle’s medium (DMEM; Thermo Fisher Scientific) supplemented with 10% (vol/vol) fetal bovine serum, 1% penicillin-streptomycin (working concentration of 50 µg/mL–50 U/mL, respectively), and G418-geneticin (working concentration 500 µg/mL). H358 cells (bronchioalveolar carcinoma cell line, ATCC # CRL-5807) were maintained at 37°C and 5% CO_2_, in Roswell Park Memorial Institute 1640 medium (RPMI 1640; Thermo Fisher Scientific) supplemented with 10% fetal bovine serum (vol/vol), 1% penicillin-streptomycin, 2 mM GlutaMax, 10 mM HEPES pH 7.2–7.5, 1 mM sodium pyruvate, and nonessential amino acids. HeLa cells (ATCC # CCL-2) were maintained at 37°C and 5% CO_2_, in DMEM supplemented with 10% (vol/vol) fetal bovine serum, and 1% penicillin-streptomycin (working concentration of 50 µg/mL and 50 U/mL, respectively).

### Generation of MeV-MFA^tracker^

mCherry sequence was amplified from pCG-mCherryNLS-H using primers 19 and 20 incorporating PacI and SpeI restriction sites (60). The amplified mCherry sequence was transferred into pCG-Hwt using overlapping PCR amplified via primer pair 21 and 22 to generate pCG-mCherryHwt. PacI and SpeI were used to introduce the mCherryH fragment from pCG-mCherryH into p(+)MV323(mCherryNLS)H, generating p(+)MV323mCherryH, hereby termed as MeV-MFA^tracker^.

### Generation of MeV^Dual-Tracker^ virus

pCDNA3_UnaG-Flag-Sec61B was gifted by Dr. Hyun-Woo Rhee (Addgene plasmid # 83413). UnaG sequence was amplified from this plasmid using primers 1 and 2 (Su pCG-H/XhEGF was used for generation of intermediate plasmid pCG-H/XUnaG; Table S2) (61). Briefly, pCG-H/XhEGF was digested by PvuII and SpeI to transfer the XhEGF segment to pCG-Hwt. Site-directed mutagenesis was performed to incorporate BsiWI and NotI sites in pCG-H/XhEGF using primer pairs 5,6 and 7,8. pCG-H/XhEGF and amplified UnaG sequence from pCDNA3_UnaG-Flag-Sec61B were digested with BsiWI and NotI and ligated, generating pCG-H/XUnaG. Primer pairs 3 and 4 were used to change the second amino acid of UnaG from valine to leucine to enhance fluorescence, generating pCG-H/XeUnaG (e denotes enhanced UnaG) (62). Plasmid coding for full-length MeV genome in IC323 background, now referred to as p(+)MV323H/XeUnaG, was generated by transferring PacI and SpeI digested fragment from pCG-H/XeUnaG to p(+)MV323(mCherryNLS)H (60). Site-directed mutagenesis was performed on pCG-H/X-eUnaG and pCG-GFP-P to incorporate MluI and AatII, respectively, using primer pairs 11,12 and 13,14 (63). mCherry sequence was amplified from pCG-mCherry-H using primers 9 and 10 and ligated into pCG-GFP-P by removing GFP sequence using enzymes MluI and AatII, generating pCG-mCherry-P (60). Overlap extension PCR was performed with primer pairs 12 and 13 first, and 11 and 14 later, to integrate MluI site and intergenic sequence (IGS) before P protein in pCG-H/X-eUnaG, generating pCG-H/X-eUnaG(IGS). PacI and SpeI were used to introduce the H/X-eUnaG(IGS) fragment from pCG-H/X-eUnaG(IGS) into p(+)MV323(mCherryNLS)H, generating p(+)MV323H/XeUnaG(IGS). MluI and SpeI digested fragment from pCG-mCherry-P was ligated into p(+)MV323H/XeUnaG, generating p(+)MV323H/XeUnaG-mCherryP, termed “MeV^Dual-Tracker^.”

We were unable to detect UnaG fluorescence in HAE but could detect fluorescence in H358 cells. We attribute this discrepancy to the presence or absence of bilirubin in the culture media. Bilirubin is a required co-factor for UnaG fluorescence and is normally present in serum. Unlike H358 cells, HAE are maintained in serum-free media. Indeed, the presence of serum results in de-differentiation of well-differentiated primary cultures, which results in artifactual MeV expression (11).

### Virus production, propagation, and titer determination

All recombinant MeV used in this study are derivatives of wild-type strain IC-323 (16). MeV-GFP, MeV-nYFP, and MeV-RNP^tracker^ were generated as previously published (15, 28). MeV-MFA^tracker^ and MeV^Dual-Tracker^ viruses were rescued from single clones to form passage 1 stocks. All MeV stocks were amplified and titrated via TCID_50_ in Vero-hSLAMF1 cells, as previously described (15, 16).

### Viral vector production and transduction

Vectors were produced by the University of Iowa Viral Vector Core (https://medicine.uiowa.edu/vectorcore). Whole plasmid sequencing was performed by Plasmidsaurus. Constructs were synthesized based on deposited sequences (Table S3) and cloned into Ad production shuttles by GenScript. Ad5-CMV-eGFP was produced, purified, and titered as previously described (35). Ad-MeV-Hemagglutinin-mTagBFP2, Ad-MeV-Hemagglutinin-Y543A-mTagBFP2, Ad-MeV-Fusion-mCherry, Ad-MeV-Matrix-mCitrine, Ad-MeV-MFH, Ad-NiV-Glycoprotein-mTagBFP2, and Ad-NiV-Fusion-mCherry were produced, and vector titers were determined by flow cytometry using fluorescent reporter expression and limiting dilutions. To transduce airway epithelial cells, 50 µL vector inoculum at MOI 250, including the Ad vector and 4.5% NaCl, was applied apically for 2 h (36). Ad-MeV-MFH was generated with a helper-dependent Ad5 vector backbone to accommodate a larger cassette size. All other vectors were generated on a replication-defective first-generation Ad5 vector backbone. For constructs with doxycycline-inducible promoters, 1 µg/mL doxycycline was added to the basolateral media and refreshed every 3–5 days. To generate Y543A point mutant, the plasmid backbone of Ad-MeV-Hemagglutinin-mTagBFP2 was mutagenized with Q5 Site-Directed Mutagenesis Kit (NEB Cat #E0554S), according to the manufacturer’s instructions. Primer set 23 and 24 were utilized for generating a Y543A mutation in Ad-H (Table S2).

### Infection of HAE and immortalized cells

HAE cultures were infected with MeV as previously published (5, 11, 17, 27-29). Briefly, HAE cultures were inverted to expose the basolateral surface. Virus diluted in 80 µL RPMI 1640 supplemented with 10% fetal bovine serum (vol/vol), 1% penicillin-streptomycin, and 0.05 mM 2-mercaptoethanol was applied to the inverted Transwell and incubated for 3–4 h at 37°C under 5% CO_2_. After the infection, the inoculum was removed, and the cultures were returned upright with medium in the basolateral chamber. H358 cells plated onto six-well tissue culture-treated plates (Corning) at a density of 300,000 cells/well and incubated overnight. Cells were infected for 2 h in Eagle’s minimum essential medium (Opti-MEM, Thermo Fisher Scientific; #31985062) before inoculum was removed and the culture medium replaced.

### Immunostaining and microscopy

Cells were prepared for immunostaining by fixing with 4% paraformaldehyde for 15 min. Cells were then blocked and permeabilized in Pierce SuperBlock (Thermo Fisher Scientific) containing 0.1%–0.2% Triton X-100 at room temperature for 1 h. Primary antibody was incubated in permeabilization buffer, rocking overnight at 4°C, and secondary antibody was incubated in permeabilization buffer for 1 h at room temperature. To stain for F-actin, HAE were incubated with Phalloidin-Alexa 647 (1:400 in PBS, #A22287; Thermo Fisher Scientific) for 30 min at room temperature. HAE transwell filters were then cut out from the transwell insert and mounted on glass microscope slides using VECTASHIELD Mounting Medium with DAPI (H-1200-10; Vector Laboratories, Inc., Burlingame, CA) or without DAPI (H-1000-10) and sealed using clear nail polish. Confocal images were captured on either a Leica TCS SP3 (Leica Microsystems), a Leica SP8 (Leica Microsystems), or an Olympus FluoView V1000 (Olympus) confocal microscope, all with a 63× objective, as noted in figure legends. Live microscopy was performed with an ECHO spinning disk confocal (ECHO, San Diego, CA). Image processing was performed using ImageJ2 version 2.4.41. Primary antibodies were used at 1:100 dilution (Table S4). Polyclonal rabbit serum was utilized against MeV Hemagglutinin or MeV Phosphoprotein as previously described (9). Secondary antibodies used at dilution 1:1,000 include: donkey-anti-mouse 647 (#A31571), goat anti-mouse 647 (#A21235), goat anti-mouse 405 (#A31553), goat anti-mouse 546 (#A11003), and goat anti-rabbit 647 (#A21244). For live imaging of F-actin, 1 μM SiR-Actin 647 (Spirochrome; #CY-SC001) and 10 μM verapamil were added to cell media throughout imaging, according to the manufacturer’s instructions.

### MeV RDRP inhibitor

HAE were infected with MeV-eGFP at an MOI of 1 using the standard protocol. At progressing timepoints post infection, 50 μM of RNA-dependent RNA polymerase inhibitor, ERDRP-0159 (MedChemExpress; #HY-102074), or an equivalent volume of DMSO (mock) was added at different timepoints to the basolateral media from 1 to 5 dpi. HAE were imaged daily from 3 to 7 dpi on an All-in-one-Fluorescence Microscope BZ-X800E (Keyence Corporation of America) and analyzed in Fiji for infectious center size.

### Quantification of cytoskeleton breakdown

Image acquisition was performed using an All-in-one-Fluorescence Microscope BZ-X800E with a 40× objective. Five representative images of each fixed culture stained for F-actin were taken, blinded to vector fluorophore fluorescence. Disrupted F-actin was measured using the freehand tool in ImageJ2 version 2.4.41. F-actin depolymerization was determined by accumulating the area measurements of all regions where loss of F-actin is visualized in the image frame. These data were plotted by area in GraphPad Prism. This measurement was repeated with 5–6 different human donors.

### Immunoblot

H358 or HeLa cells were mock infected, infected with MeV-GFP at an MOI of 0.1 for 2 h, or transduced with Ad-based vector at an MOI of 50 for 2 h in duplicate in six-well plates. After 2–3 days post infection/transduction, cells were lysed in RIPA Lysis and Extraction Buffer (Thermo Fisher Scientific, #89900), and duplicate wells were combined to concentrate samples. Lysed buffer was supplemented with cOmplete, Mini EDTA-free Protease Inhibitor Cocktail (Millipore Sigma, Darmstadt, Germany, #11836170001). Samples were freeze-thawed before Laemmli buffer was added, and samples were boiled at 95°C for 5 min. A total of 50 µg of protein sample was loaded into a 4%–20% Mini-PROTEAN TGX Precast Protein Gel (Bio-Rad, Hercules, CA, #4561094). Protein was run on gels at 100 V for 30–45 min then transferred onto polyvinylidene difluoride (PVDF) membranes (Immobilon-FL, Millipore; IPFL 00010) at 100 V for 1 h at 4°C. Membranes were blocked with 1× TBS buffer supplemented with 0.1% Tween and 5% milk for 1 h at room temperature. Primary antibodies were used at dilution 1:500 (Table S4). Polyclonal rabbit serum against the cytoplasmic tail domains of MeV Hemagglutinin or MeV Fusion was used as previously described (9). Secondary antibodies were used at 1:5,000 dilution conjugated to HRP including goat anti-rabbit HRP (Thermo; #31460) and goat anti-mouse HRP (Thermo; #31432). Blots were developed with SuperSignal West Pico PLUS Chemiluminescent Substrate (Thermo Fisher Scientific, #34580).

### Statistical analyses

Unless stated otherwise, each study was performed with at least three donors. Statistical analyses are specified in each figure legend. Significant values are shown by asterisks: *P < 0.05; **P < 0.01; ***P < 0.001; ****P < 0.0001, or ns (not significant).

## Data Availability

The authors confirm that the data supporting the findings of this study are available within the article or its supplemental materials.

## SUPPLEMENTAL MATERIAL

Supplemental figures and tables spectrum.02297-26-s0001.pdf Fig. S1 to S5 and Tables S1 to S4.

Movie S1 spectrum.02297-26-s0002.avi Live cell imaging of spread via RNPtracker. HAE were infected with MeV-RNPtracker at MOI 1 for 4 h. The time-lapse movie started at 24 hpi, when single-infected cells are visible by eGFP fluorescence. Images were collected every 0.5 h for 72 h. During imaging, cells were maintained at 37°C, 5% CO_2_.

Movie S2 spectrum.02297-26-s0003.mov Live cell imaging of spread via MFAtracker. HAE were infected with MeV-MFAtracker at MOI 1 for 4 h. The time-lapse movie started at 24 hpi, when single infected cells are visible by mCherry fluorescence. Images were collected every 0.5 h for 72 h. During imaging, cells were maintained at 37°C, 5% CO_2_.

Movie S3 spectrum.02297-26-s0004.mov Live cell imaging of MeVDual-Tracker cell-to-cell spread. H358 cells were infected with MeVDual-Tracker at MOI 0.1 for 2 h. At 23 hpi, cells were incubated in media with 1 µM SiR-Actin 647 and incubated at least 1 h before start of live imaging. Time-lapse movie started at 24 hpi. Images were collected every 0.5 h for 72 h. During imaging, cells were maintained at 37°C, 5% CO_2_. Scale bar = 200 µm.

Movie S4 spectrum.02297-26-s0005.avi Live-cell imaging of Ad-MFH induced cytoplasmic sharing. HAE were transduced with Ad-MFH at MOI 50 for 2 h to the apical surface. After singly transduced cells are visible by eGFP fluorescence at 2 dpt, 1 µg/mL doxycycline was added to the basolateral media to start the expression of MeV proteins. Images were collected every 20 min for 72 h. During imaging, cells were maintained at 37°C, 5% CO_2_.

Movie S5 spectrum.02297-26-s0006.avi Live cell imaging of spread via WT MeV-eGFP. HAE were infected with MeV-GFP for 4 h at MOI 1. Movie started at 24 hpi, when single infected cells are visible via GFP fluorescence. Visible points were selected via ECHO software and images were taken every 30 min for 72 h. During imaging, cells were maintained at 37°C, 5% CO_2_. Scale bar = 200 µm.

ASM does not own the copyrights to Supplemental Material that may be linked to, or accessed through, an article. The authors have granted ASM a non-exclusive, world-wide license to publish the Supplemental Material files. Please contact the corresponding author directly for reuse.

## References

[1] Guerra FM, Bolotin S, Lim G, Heffernan J, Deeks SL, Li Y, Crowcroft NS. 2017. The basic reproduction number (R0) of measles: a systematic review. Lancet Infect Dis 17:e420–e428. doi:10.1016/S1473-3099(17)30307-9 28757186

[2] Rima BK, Duprex WP. 2009. The measles virus replication cycle. Curr Top Microbiol Immunol 329:77–102. doi:10.1007/978-3-540-70523-9_5 19198563

[3] Zhou H, Sun Y, Guo Y, Lou Z. 2013. Structural perspective on the formation of ribonucleoprotein complex in negative-sense single-stranded RNA viruses. Trends Microbiol 21:475–484. doi:10.1016/j.tim.2013.07.006 23953596

[4] Luo M, Terrell JR, Mcmanus SA. 2020. Nucleocapsid structure of negative strand RNA virus. Viruses 12:835. doi:10.3390/v12080835 PMC7472042 32751700

[5] Singh BK, Hornick AL, Krishnamurthy S, Locke AC, Mendoza CA, Mateo M, Miller-Hunt CL, Cattaneo R, Sinn PL. 2015. The Nectin-4/Afadin protein complex and intercellular membrane pores contribute to rapid spread of measles virus in primary human airway epithelia. J Virol 89:7089–7096. doi:10.1128/JVI.00821-15 PMC4473566 25926640

[6] Ke Z, Strauss JD, Hampton CM, Brindley MA, Dillard RS, Leon F, Lamb KM, Plemper RK, Wright ER. 2018. Promotion of virus assembly and organization by the measles virus matrix protein. Nat Commun 9:1736. doi:10.1038/s41467-018-04058-2 PMC5928126 29712906

[7] Cathomen T, Mrkic B, Spehner D, Drillien R, Naef R, Pavlovic J, Aguzzi A, Billeter MA, Cattaneo R. 1998. A matrix-less measles virus is infectious and elicits extensive cell fusion: consequences for propagation in the brain. EMBO J 17:3899–3908. doi:10.1093/emboj/17.14.3899 PMC1170725 9670007

[8] Navaratnarajah CK, Generous AR, Yousaf I, Cattaneo R. 2020. Receptor-mediated cell entry of paramyxoviruses: Mechanisms, and consequences for tropism and pathogenesis. J Biol Chem 295:2771–2786. doi:10.1074/jbc.REV119.009961 PMC7049954 31949044

[9] Cathomen T, Naim HY, Cattaneo R. 1998. Measles viruses with altered envelope protein cytoplasmic tails gain cell fusion competence. J Virol 72:1224–1234. doi:10.1128/JVI.72.2.1224-1234.1998 PMC124600 9445022

[10] Iwasaki M, Takeda M, Shirogane Y, Nakatsu Y, Nakamura T, Yanagi Y. 2009. The matrix protein of measles virus regulates viral RNA synthesis and assembly by interacting with the nucleocapsid protein. J Virol 83:10374–10383. doi:10.1128/JVI.01056-09 PMC2753126 19656884

[11] Kaufman JW, Singh BK, Durnell LA, Sinn PL. 2023. Representative measles virus infection requires appropriate airway epithelia culture conditions. J Virol 97:e0105123. doi:10.1128/jvi.01051-23 PMC10617594 37732787

[12] Karp PH, Moninger TO, Weber SP, Nesselhauf TS, Launspach JL, Zabner J, Welsh MJ. 2002. An *in vitro* model of differentiated human airway epithelia. methods for establishing primary cultures. Methods Mol Biol 188:115–137. doi:10.1385/1-59259-185-X:115 11987537

[13] Pezzulo AA, Starner TD, Scheetz TE, Traver GL, Tilley AE, Harvey BG, Crystal RG, McCray PB, Zabner J. 2011. The air-liquid interface and use of primary cell cultures are important to recapitulate the transcriptional profile of in vivo airway epithelia. Am J Physiol Lung Cell Mol Physiol 300:L25–31. doi:10.1152/ajplung.00256.2010 PMC3023285 20971803

[14] Sinn PL, Williams G, Vongpunsawad S, Cattaneo R, McCray PB Jr. 2002. Measles virus preferentially transduces the basolateral surface of well-differentiated human airway epithelia. J Virol 76:2403–2409. doi:10.1128/jvi.76.5.2403-2409.2002 PMC153805 11836418

[15] Leonard VHJ, Sinn PL, Hodge G, Miest T, Devaux P, Oezguen N, Braun W, McCray PB Jr, McChesney MB, Cattaneo R. 2008. Measles virus blind to its epithelial cell receptor remains virulent in rhesus monkeys but cannot cross the airway epithelium and is not shed. J Clin Invest 118:2448–2458. doi:10.1172/JCI35454 PMC2430500 18568079

[16] Takeda M, Takeuchi K, Miyajima N, Kobune F, Ami Y, Nagata N, Suzaki Y, Nagai Y, Tashiro M. 2000. Recovery of pathogenic measles virus from cloned cDNA. J Virol 74:6643–6647. doi:10.1128/jvi.74.14.6643-6647.2000 PMC112175 10864679

[17] Hippee CE, Singh BK, Thurman AL, Cooney AL, Pezzulo AA, Cattaneo R, Sinn PL. 2021. Measles virus exits human airway epithelia within dislodged metabolically active infectious centers. PLoS Pathog 17:e1009458. doi:10.1371/journal.ppat.1009458 PMC8384213 34383863

[18] Frenzke M, Sawatsky B, Wong XX, Delpeut S, Mateo M, Cattaneo R, von Messling V. 2013. Nectin-4-dependent measles virus spread to the cynomolgus monkey tracheal epithelium: role of infected immune cells infiltrating the lamina propria. J Virol 87:2526–2534. doi:10.1128/JVI.03037-12 PMC3571369 23255790

[19] Mühlebach MD, Mateo M, Sinn PL, Prüfer S, Uhlig KM, Leonard VHJ, Navaratnarajah CK, Frenzke M, Wong XX, Sawatsky B, Ramachandran S, McCray PB, Cichutek K, von Messling V, Lopez M, Cattaneo R. 2011. Adherens junction protein nectin-4 is the epithelial receptor for measles virus. Nature 480:530–533. doi:10.1038/nature10639 PMC3245798 22048310

[20] Pratakpiriya W, Seki F, Otsuki N, Sakai K, Fukuhara H, Katamoto H, Hirai T, Maenaka K, Techangamsuwan S, Lan NT, Takeda M, Yamaguchi R. 2012. Nectin4 is an epithelial cell receptor for canine distemper virus and involved in neurovirulence. J Virol 86:10207–10210. doi:10.1128/JVI.00824-12 PMC3446623 22761370

[21] Noyce RS, Bondre DG, Ha MN, Lin LT, Sisson G, Tsao MS, Richardson CD. 2011. Tumor cell marker PVRL4 (nectin 4) is an epithelial cell receptor for measles virus. PLoS Pathog 7:e1002240. doi:10.1371/journal.ppat.1002240 PMC3161989 21901103

[22] Noyce RS, Delpeut S, Richardson CD. 2013. Dog nectin-4 is an epithelial cell receptor for canine distemper virus that facilitates virus entry and syncytia formation. Virology 436:210–220. doi:10.1016/j.virol.2012.11.011 23260107

[23] Navaratnarajah CK, Negi S, Braun W, Cattaneo R. 2012. Membrane fusion triggering: three modules with different structure and function in the upper half of the measles virus attachment protein stalk. J Biol Chem 287:38543–38551. doi:10.1074/jbc.M112.410563 PMC3493899 23007387

[24] Navaratnarajah CK, Kumar S, Generous A, Apte-Sengupta S, Mateo M, Cattaneo R. 2014. The measles virus hemagglutinin stalk: structures and functions of the central fusion activation and membrane-proximal segments. J Virol 88:6158–6167. doi:10.1128/JVI.02846-13 PMC4093900 24648460

[25] Navaratnarajah CK, Leonard VHJ, Cattaneo R. 2009. Measles virus glycoprotein complex assembly, receptor attachment, and cell entry. Curr Top Microbiol Immunol 329:59–76. doi:10.1007/978-3-540-70523-9_4 PMC7121846 19198562

[26] Plemper RK, Brindley MA, Iorio RM. 2011. Structural and mechanistic studies of measles virus illuminate paramyxovirus entry. PLoS Pathog 7:e1002058. doi:10.1371/journal.ppat.1002058 PMC3107210 21655106

[27] Singh BK, Li N, Mark AC, Mateo M, Cattaneo R, Sinn PL. 2016. Cell-to-cell contact and nectin-4 govern spread of measles virus from primary human myeloid cells to primary human airway epithelial cells. J Virol 90:6808–6817. doi:10.1128/JVI.00266-16 PMC4944272 27194761

[28] Singh BK, Pfaller CK, Cattaneo R, Sinn PL. 2019. Measles virus ribonucleoprotein complexes rapidly spread across well-differentiated primary human airway epithelial cells along f-actin rings. mBio 10:e02434-19. doi:10.1128/mBio.02434-19 PMC6879720 31772054

[29] Hippee CE, Durnell LA, Kaufman JW, Murray E, Singh BK, Sinn PL. 2025. Epithelial-to-mesenchymal transition and live cell extrusion contribute to measles virus release from human airway epithelia. J Virol 99:e0122024. doi:10.1128/jvi.01220-24 PMC11852777 39791903

[30] Durnell LA, Hippee CE, Cattaneo R, Bartlett JA, Singh BK, Sinn PL. 2023. Interferon-independent processes constrain measles virus cell-to-cell spread in primary human airway epithelial cells. Microbiol Spectr 11:e0136123. doi:10.1128/spectrum.01361-23 PMC10580916 37724882

[31] Wang Y, Zhao L, Zhang Y, Gao X, Wang Y, Shi W, Kornberg RD, Zhang H. 2025. Structures of the measles virus polymerase complex with non-nucleoside inhibitors and mechanism of inhibition. Cell 188:4913–4923. doi:10.1016/j.cell.2025.06.017 40628260

[32] Wittwer K, Anderson DE, Pfeffermann K, Cox RM, Wolf JD, Santibanez S, Mankertz A, Plesker R, Sticher ZM, Kolkykhalov AA, Natchus MG, Pfaller CK, Plemper RK, von Messling V. 2021. Small-molecule polymerase inhibitor protects non-human primates from measles and reduces shedding. Nat Commun 12:5233. doi:10.1038/s41467-021-25497-4 PMC8413292 34475387

[33] Cox RM, Sourimant J, Govindarajan M, Natchus MG, Plemper RK. 2021. Therapeutic targeting of measles virus polymerase with ERDRP-0519 suppresses all RNA synthesis activity. PLoS Pathog 17:e1009371. doi:10.1371/journal.ppat.1009371 PMC7935272 33621266

[34] Krumm SA, Yan D, Hovingh ES, Evers TJ, Enkirch T, Reddy GP, Sun A, Saindane MT, Arrendale RF, Painter G, Liotta DC, Natchus MG, von Messling V, Plemper RK. 2014. An orally available, small-molecule polymerase inhibitor shows efficacy against a lethal morbillivirus infection in a large animal model. Sci Transl Med 6:232ra52. doi:10.1126/scitranslmed.3008517 PMC4080709 24739760

[35] Anderson RD, Haskell RE, Xia H, Roessler BJ, Davidson BL. 2000. A simple method for the rapid generation of recombinant adenovirus vectors. Gene Ther 7:1034–1038. doi:10.1038/sj.gt.3301197 10871752

[36] Cooney AL, Loza LM, Najdawi K, Brommel CM, McCray PB, Sinn PL. 2024. High ionic strength vector formulations enhance gene transfer to airway epithelia. Nucleic Acids Res 52:9369–9383. doi:10.1093/nar/gkae640 PMC11381324 39077931

[37] Alkhatib G, Richardson C, Shen SH. 1990. Intracellular processing, glycosylation, and cell-surface expression of the measles virus fusion protein (F) encoded by a recombinant adenovirus. Virology 175:262–270. doi:10.1016/0042-6822(90)90207-8 2309445

[38] Vialard J, Lalumière M, Vernet T, Briedis D, Alkhatib G, Henning D, Levin D, Richardson C. 1990. Synthesis of the membrane fusion and hemagglutinin proteins of measles virus, using a novel baculovirus vector containing the beta-galactosidase gene. J Virol 64:37–50. doi:10.1128/JVI.64.1.37-50.1990 PMC249037 2104544

[39] Müller M, Fischer K, Woehnke E, Zaeck LM, Prönnecke C, Knittler MR, Karger A, Diederich S, Finke S. 2023. Analysis of nipah virus replication and host proteome response patterns in differentiated porcine airway epithelial cells cultured at the air-liquid interface. Viruses 15:961. doi:10.3390/v15040961 PMC10143807 37112941

[40] Mateo M, Generous A, Sinn PL, Cattaneo R. 2015. Connections matter--how viruses use cell–cell adhesion components. J Cell Sci 128:431–439. doi:10.1242/jcs.159400 PMC4311127 26046138

[41] Cifuentes-Muñoz N, Dutch RE, Cattaneo R. 2018. Direct cell-to-cell transmission of respiratory viruses: The fast lanes. PLoS Pathog 14:e1007015. doi:10.1371/journal.ppat.1007015 PMC6023113 29953542

[42] Bonaparte MI, Dimitrov AS, Bossart KN, Crameri G, Mungall BA, Bishop KA, Choudhry V, Dimitrov DS, Wang LF, Eaton BT, Broder CC. 2005. Ephrin-B2 ligand is a functional receptor for Hendra virus and Nipah virus. Proc Natl Acad Sci USA 102:10652–10657. doi:10.1073/pnas.0504887102 PMC1169237 15998730

[43] Kalluri R, Weinberg RA. 2009. The basics of epithelial-mesenchymal transition. J Clin Invest 119:1420–1428. doi:10.1172/JCI39104 PMC2689101 19487818

[44] Wee K, Hediyeh-Zadeh S, Duszyc K, Verma S, N Nanavati B, Khare S, Varma A, Daly RJ, Yap AS, Davis MJ, Budnar S. 2020. Snail induces epithelial cell extrusion by regulating RhoA contractile signalling and cell-matrix adhesion. J Cell Sci 133:jcs235622. doi:10.1242/jcs.235622 32467325

[45] Le Bras GF, Taubenslag KJ, Andl CD. 2012. The regulation of cell-cell adhesion during epithelial-mesenchymal transition, motility and tumor progression. Cell Adh Migr 6:365–373. doi:10.4161/cam.21326 PMC3478259 22796940

[46] Ohsawa S, Vaughen J, Igaki T. 2018. Cell extrusion: a stress-responsive force for good or evil in epithelial homeostasis. Dev Cell 44:284–296. doi:10.1016/j.devcel.2018.01.009 PMC6207186 29408235

[47] Rosenblatt J, Raff MC, Cramer LP. 2001. An epithelial cell destined for apoptosis signals its neighbors to extrude it by an actin- and myosin-dependent mechanism. Curr Biol 11:1847–1857. doi:10.1016/s0960-9822(01)00587-5 11728307

[48] Shirogane Y, Takeda M, Tahara M, Ikegame S, Nakamura T, Yanagi Y. 2010. Epithelial-mesenchymal transition abolishes the susceptibility of polarized epithelial cell lines to measles virus. J Biol Chem 285:20882–20890. doi:10.1074/jbc.M110.102590 PMC2898294 20435897

[49] Wakimoto H, Shimodo M, Satoh Y, Kitagawa Y, Takeuchi K, Gotoh B, Itoh M. 2013. F-actin modulates measles virus cell-cell fusion and assembly by altering the interaction between the matrix protein and the cytoplasmic tail of hemagglutinin. J Virol 87:1974–1984. doi:10.1128/JVI.02371-12 PMC3571460 23221571

[50] Riedl P, Moll M, Klenk HD, Maisner A. 2002. Measles virus matrix protein is not cotransported with the viral glycoproteins but requires virus infection for efficient surface targeting. Virus Res 83:1–12. doi:10.1016/s0168-1702(01)00379-3 11864737

[51] Lobanova LM, Baig TT, Tikoo SK, Zakhartchouk AN. 2010. Mucosal adenovirus-vectored vaccine for measles. Vaccine 28:7613–7619. doi:10.1016/j.vaccine.2010.09.055 20887832

[52] Alkhatib G, Briedis DJ. 1988. High-level eucaryotic in vivo expression of biologically active measles virus hemagglutinin by using an adenovirus type 5 helper-free vector system. J Virol 62:2718–2727. doi:10.1128/JVI.62.8.2718-2727.1988 PMC253705 3292790

[53] Fooks AR, Jeevarajah D, Lee J, Warnes A, Niewiesk S, ter Meulen V, Stephenson JR, Clegg JC. 1998. Oral or parenteral administration of replication-deficient adenoviruses expressing the measles virus haemagglutinin and fusion proteins: protective immune responses in rodents. J Gen Virol 79:1027–1031. doi:10.1099/0022-1317-79-5-1027 9603317

[54] Nielsen IH, Rovsing AB, Janns JH, Thomsen EA, Ruzo A, Bøggild A, Nedergaard F, Møller CT, Boesen T, Degn SE, Shah JV, Mikkelsen JG. 2024. Cell-targeted gene modification by delivery of CRISPR-Cas9 ribonucleoprotein complexes in pseudotyped lentivirus-derived nanoparticles. Mol Ther Nucleic Acids 35:102318. doi:10.1016/j.omtn.2024.102318 PMC11426049 39329149

[55] Ayala-Breton C, Russell LOJ, Russell SJ, Peng KW. 2014. Faster replication and higher expression levels of viral glycoproteins give the vesicular stomatitis virus/measles virus hybrid VSV-FH a growth advantage over measles virus. J Virol 88:8332–8339. doi:10.1128/JVI.03823-13 PMC4135973 24829351

[56] Winter N, Kolbe C, Ferdos S, Steiner L, Bartelsen N, Lock D, Engert F, Engels B, Assenmacher M, Schindler M, Lauer UM, Schaser T, Cordes N. 2025. A novel pseudotype derived of the canine distemper virus for adapter-mediated lentiviral transduction *in vivo*. Mol Ther Methods Clin Dev 33:101526. doi:10.1016/j.omtm.2025.101526 PMC12305328 40734704

[57] Palmer DJ, Ng P. 2005. Helper-dependent adenoviral vectors for gene therapy. Hum Gene Ther 16:1–16. doi:10.1089/hum.2005.16.1 15703484

[58] Plattet P, Alves L, Herren M, Aguilar HC. 2016. Measles virus fusion protein: structure, function and inhibition. Viruses 8:112. doi:10.3390/v8040112 PMC4848605 27110811

[59] Ono N, Tatsuo H, Hidaka Y, Aoki T, Minagawa H, Yanagi Y. 2001. Measles viruses on throat swabs from measles patients use signaling lymphocytic activation molecule (CDw150) but not CD46 as a cellular receptor. J Virol 75:4399–4401. doi:10.1128/JVI.75.9.4399-4401.2001 PMC114185 11287589

[60] Donohue RC, Pfaller CK, Cattaneo R. 2019. Cyclical adaptation of measles virus quasispecies to epithelial and lymphocytic cells: To V, or not to V. PLoS Pathog 15:e1007605. doi:10.1371/journal.ppat.1007605 PMC6395005 30768648

[61] Schneider U, Bullough F, Vongpunsawad S, Russell SJ, Cattaneo R. 2000. Recombinant measles viruses efficiently entering cells through targeted receptors. J Virol 74:9928–9936. doi:10.1128/jvi.74.21.9928-9936.2000 PMC102030 11024120

[62] Lee E, Shim SH, Cho M. 2018. Fluorescence enhancement of a ligand-activated fluorescent protein induced by collective noncovalent interactions. Chem Sci 9:8325–8336. doi:10.1039/c8sc03558j PMC6249632 30542580

[63] Generous AR, Harrison OJ, Troyanovsky RB, Mateo M, Navaratnarajah CK, Donohue RC, Pfaller CK, Alekhina O, Sergeeva AP, Indra I, Thornburg T, Kochetkova I, Billadeau DD, Taylor MP, Troyanovsky SM, Honig B, Shapiro L, Cattaneo R. 2019. *Trans*-endocytosis elicited by nectins transfers cytoplasmic cargo, including infectious material, between cells. J Cell Sci 132:jcs235507. doi:10.1242/jcs.235507 PMC6737912 31331966

