# Supplementary material for: Hemagglutinin and fusion proteins precede measles virus ribonucleoproteins and marshal cell-to-cell spread in airway epithelium
Source: Microbiol Spectr. 2026 Oct 14;14(11):e02297-26. doi: 10.1128/spectrum.02297-26 (PMC13729160; doi:10.1128/spectrum.02297-26)
Supplement: Supplementary file 1 [file spectrum.02297-26-s0001.pdf]

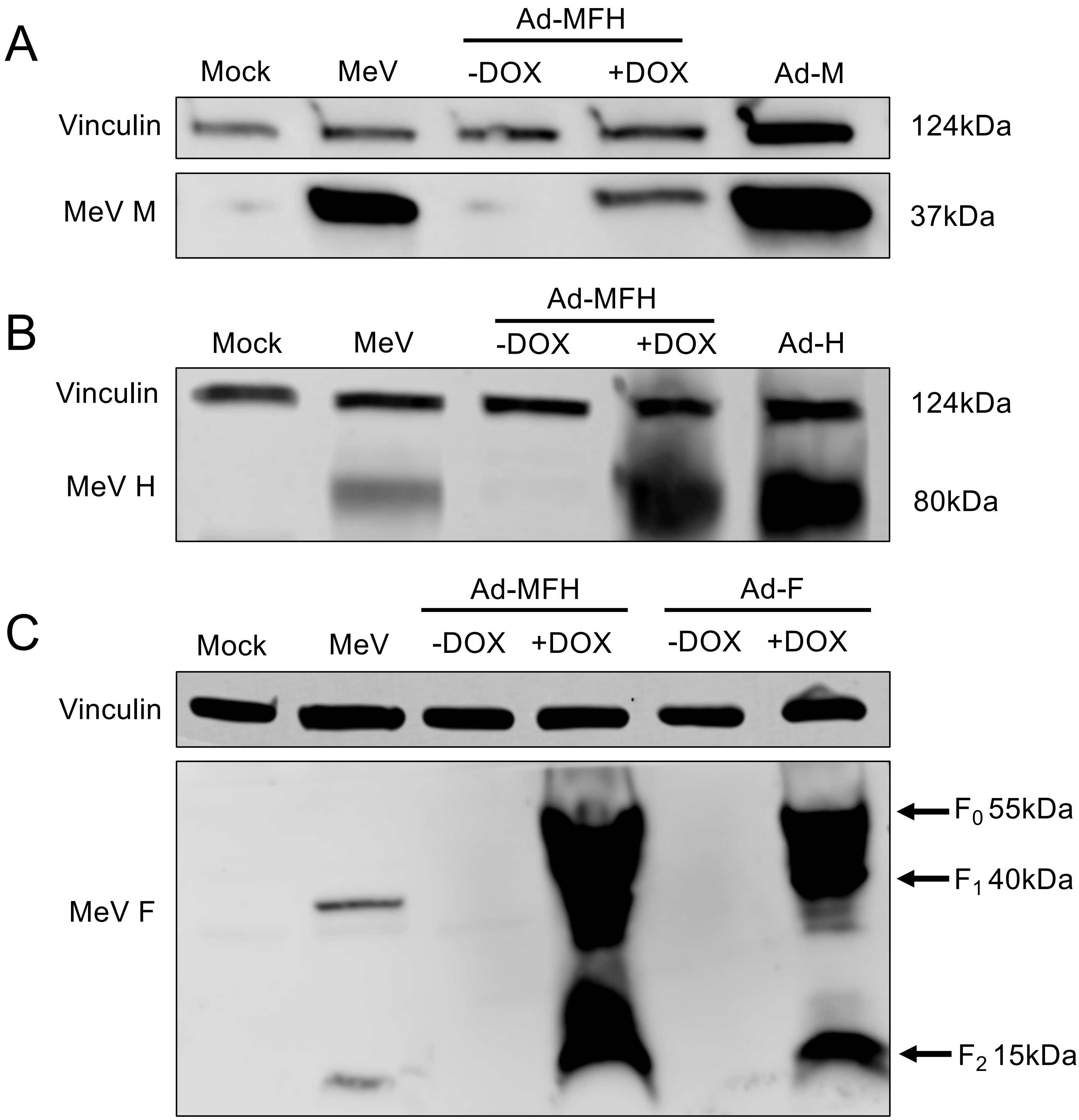

Supplemental Figure 1: Ad-MFH and Ad-H, Ad-F, and Ad-M express indicated MeV proteins. A-B) Immunoblot showing MeV proteins expressed in H358 cells transduced by Ad-M, Ad-H, or Ad-MFH vectors at MOI 50. After 2 days, lysates were collected with RIPA buffer and immunoblotted for vinculin, MeV-M, and/or MeV-H. C) Immunoblot showing MeV proteins expressed in HeLa cells transduced by Ad-F or Ad-MFH vectors at MOI 50 in the presence or absence of doxycycline. After 2 days, lysates were collected with RIPA buffer and immunoblotted for vinculin and MeV-F. The F0 precursor band was observed at approximately 55 kDa, and the active fusion subunits, F1 and F2, were seen around 40 kDa and 15 kDa, respectively.

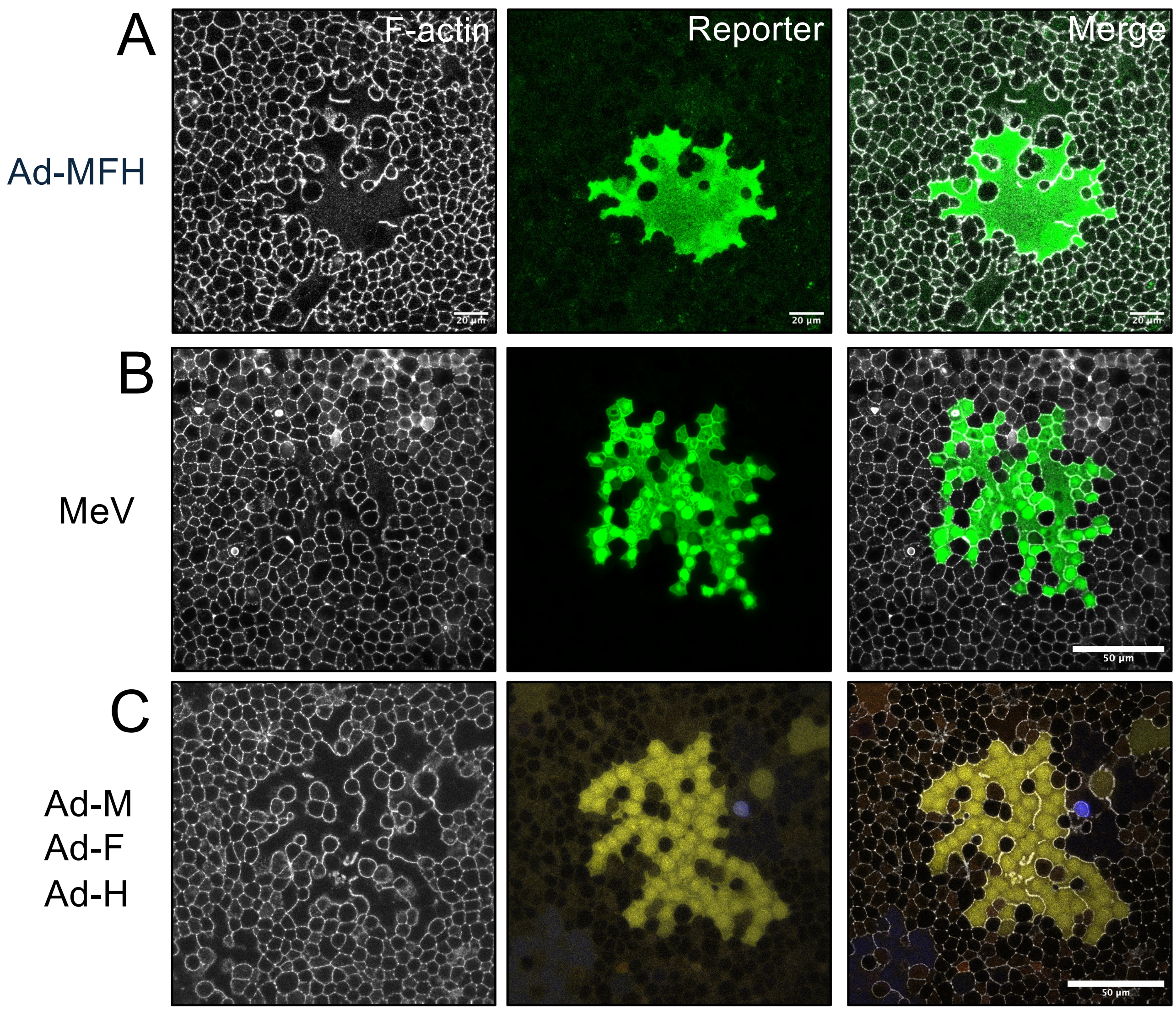

Supplemental Figure 2: Triple transduction of MeV M, F, and H results in cytoplasmic sharing resembling WT MeV. A) HAE were transduced with Ad-MFH at MOI 50 applied to the apical surface for 2 hours. After transduction, 1 mg/mL doxycycline was added in the basolateral media to induce MeV protein expression. At 5 dpt, cells were fixed, counterstained with phalloidin (white), and imaged with confocal microscopy. Scale bar = 20 μm. B) HAE were infected with MeV-GFP for 4 hours at MOI 1. Cells were fixed at 4 dpi and counterstained with phalloidin (white) and imaged with confocal microscopy. Scale bar = 50 μm. C) HAE were co-transduced with Ad-M, Ad-F, and Ad-H at MOIs 250, 50, and 250, respectively, applied to the apical surface for 2 hours. After transduction, 1 mg/mL doxycycline was added in the basolateral media for to induce MeV F expression. At 5 dpt, cells were fixed, counterstained with phalloidin (white), and imaged with confocal microscopy. Scale bar = 50 μm.

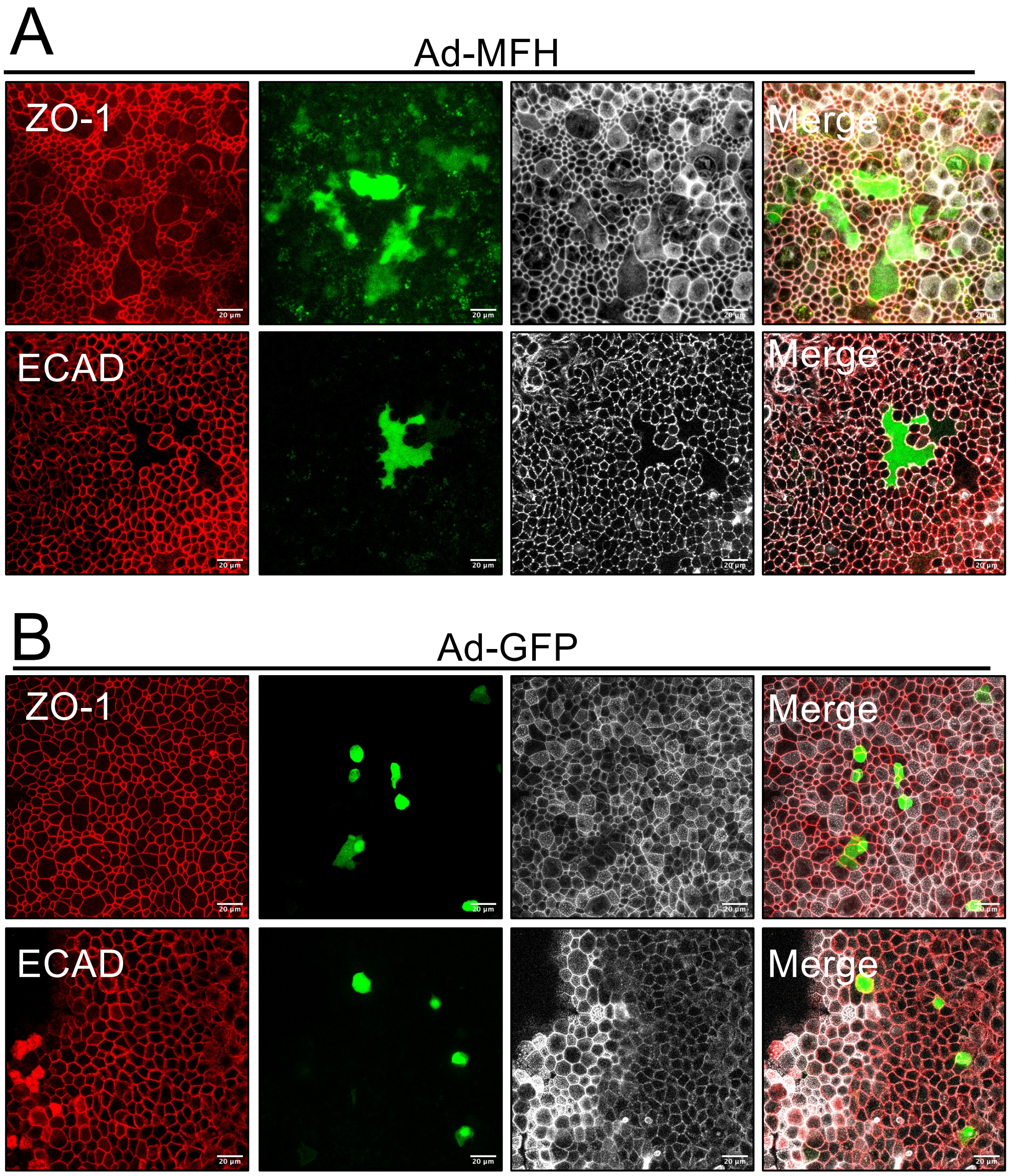

Supplemental Figure 3: Delivery of MeV H, F, and M induces epithelial to mesenchymal transition (EMT). A) HAE were transduced with Ad-MFH or (B) Ad-GFP at MOI 50 for 2 hours applied to the apical surface. 1 mg/mL doxycycline was added in the basolateral media to induce MeV protein expression. Cells were fixed at 5 dpt and counterstained with F-actin phalloidin (white) and ZO-1 or ECAD (red). Scale bar = 20 μm.

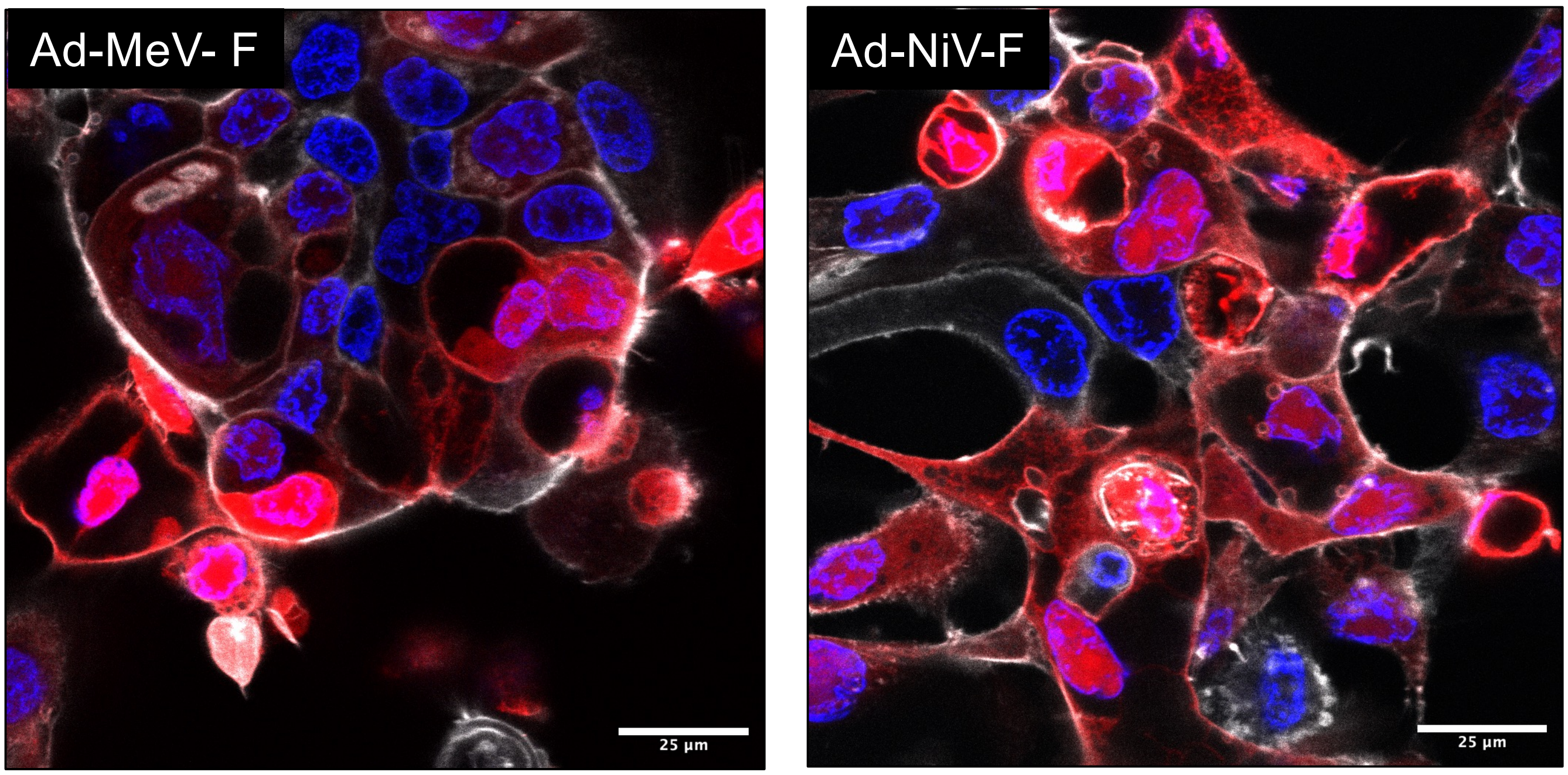

Supplemental Figure 4: Overexpression of paramyxovirus fusion protein alone is sufficient for syncytia formation in immortalized cells. H358 cells were transduced with MeV Ad-F (Figure 6B) or Ad-NiV-F (Figure 8B) in the presence of doxycycline. Expression of fusion proteins can be identified by mCherry fluorophore (red). 3 dpt, cells were fixed and stained for F-actin with phalloidin (white) and nuclei with DAPI (blue) and imaged via confocal microscopy. Scale bar = 25 µm.

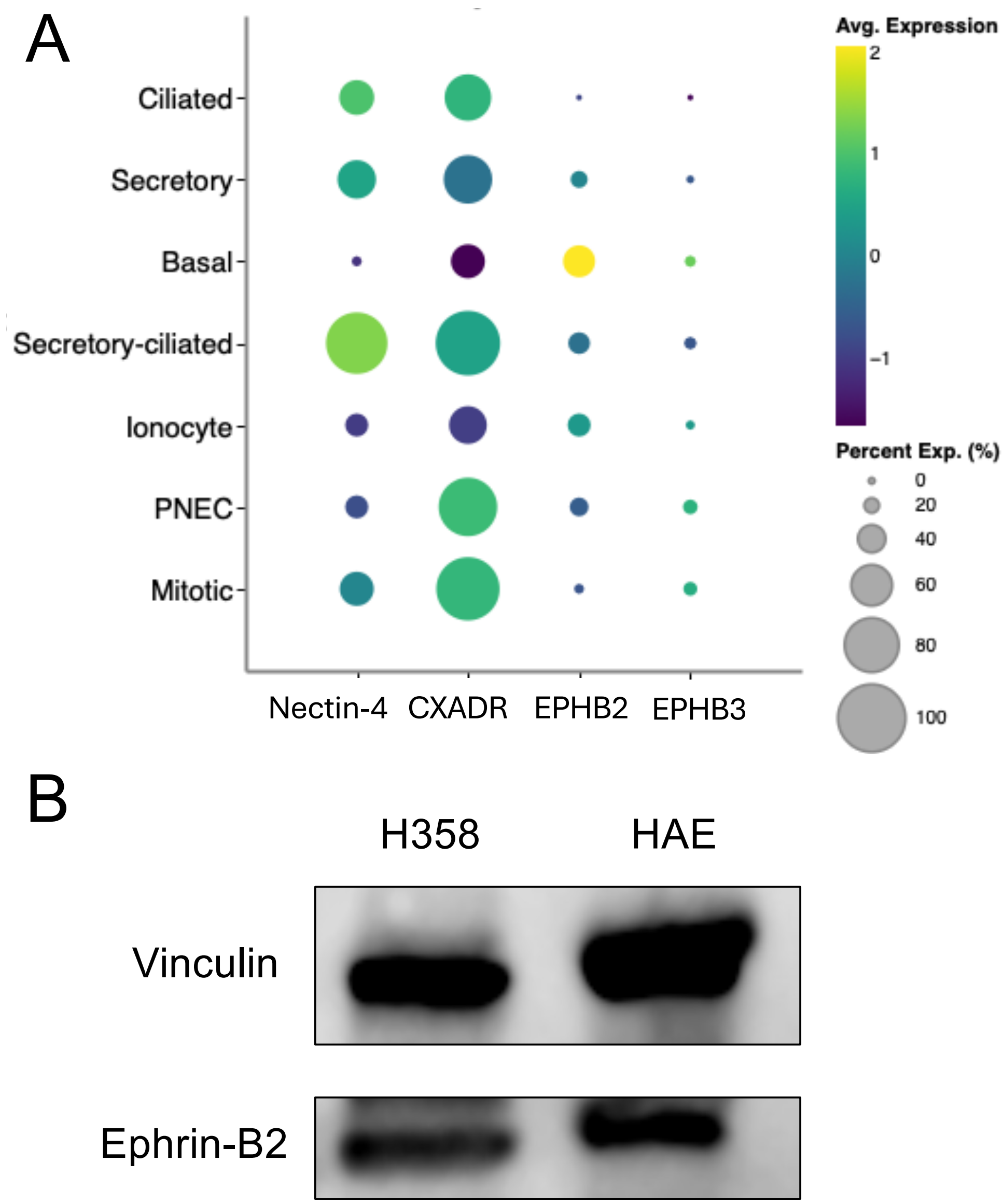

Supplemental Figure 5: Immunoblotting and scRNA-seq confirms expression of ephrin-B2 in HAE. A) Previously deposited single cell sequencing (10x Genomics) dataset performed on healthy human airway epithelia (GSE185048). Dot plot indicates absolute expression of receptors nectin-4 (MeV), CXADR (Ad5), and ephrin-B2 and ephrin-B3 (NiV) among each cell type. B) Immunoblot of uninfected H358 and HAE. Cells were lysed with RIPA buffer and immunoblotted for vinculin and ephrin-B2.

	3 dpi	4 dpi	5 dpi	6 dpi	7 dpi
Vehicle	13	22	24	21	23
L^{inhib} added 1dpi	0	0	0	0	0
L^{inhib} added 2dpi	13	12	13	13	13
L^{inhib} added 3dpi	24	24	24	24	24
L^{inhib} added 4dpi	26	27	28	31	29
L^{inhib} added 5dpi	24	24	28	33	21

Supplemental Table 1: Number of infectious centers counted for time course of L^{inhib} treatment (Figure 4). n = all infectious centers measured per time point from 2 donors

Primer Pair	Primer Name	Sequence
1	Una G FP	TAT ATA **CGT ACG** GGA GGT TCC GGA ATG GTT GAA AAA TT
2	Una G RP	TAT ATA G**GC GGC CGC** TTC GGT TGC ACG
3	eUnaG FP	CGT ACC AAC AAA TTT TTC CAG CAT CCG GAA CCT CCC G
4	eUnaG RP	CGG GAG GTT CCG GAA TGC TGG AAA AAT TTG TTG GTA CG
5	NotI FP	CAG TGT GGG ATG GTG G**GC GGC CGC** GTC AGC TGC ACA GTC AC
6	NotI RP	GTG ACT GTG CAG CTG AC**G CGG CCG C**CC ACC ATC CCA CAC TG
7	BsiWi FP	AAC CAA TAT CGA GGG AAG G**CG TAC G**CA GCC GGC CAT GGC CAA TA
8	BsiWi RP	TAT TGG CCA TGG CCG GCT G**CG TAC G**CC TTC CCT CGA TAT TGG TT
9	mCherry-P FP	AGC CCA TCA ACG GGT ATG GTG AGC AAG GGC GAG GAG GAT AAC ATG GCC
10	mCherry-P RP	TGC TCC ACC GGT ACC TCC GGA ACC TCC AGA CTT GTA CAG CTC GTC C
11	MluI FP	CGT GCA ACC GAA GCG GCC GCA TAG ACG CGT GTG TGA AAT AGA CAT CAG AAT TAA G
12	MluI RP	CTT AAT TCT GAT GTC TAT TTC ACA CA CGC GTC TAT GCG GCC GCT TCG GTT GCA CG
13	AatII FP	GCTGATGAAGATAATAATGAAGTAGGACGTCCAAGCTGATCTTTTTCCCTC
14	AatII RP	GAGGGAAAAAGATCAGCTTGGACGTCCTACTTCATTATTATCTTCATCAGC
15	Overlap-1 FP	GGA AGG CGT ACG GGA GGT TCC GGA ATG CTG GAA
16	Overlap-2 RP	GTT CAC TGG CAG CCC TAG GCG GCC GCT TCG GTT GCA C
17	Overlap-3 FP	GTG CAA CCG AAG CGG CCG CCT AGG GCT GCC AGT GAA C
18	Overlap-4 RP	CAC CAT AGG CGT TGA TGG GCT GGC GGC T
19	mCherry FP	GTAG**TTAATTAA**AACTTAGGGTGCAAGATCATCCACAATGGTGAGCAAGGGCG
20	mCherryRP	TCAC**ACTAGT**GGGTATGCCTGAT
21	mCherry-H overlap FP1	GTTCCGGAGGTACCGGTGGAATGTCACCACAACGAGACC
22	mCherry-H overlap RP1	CCGGTACCTCCGGAACCTCCCTTGTACAGCTCGTCCATGC
23	H^{Y543A} FWD	GTACTACGTGGCGTCCCCTTCTAGAAGCTTTAGC
24	H^{Y543A} REV	GCTAAAGCTTCTAGAAGGGGACGCCACGTAGTAC

Supplemental Table 2: Primer sequences for cloning and generation of $MeV^{Dual\text{-}Tracker}$, $MeV\text{-}MFA^{tracker}$, and $Ad\text{-}H^{Y543A}$.

Gene	Sequence ID	PMID
Measles Hemagglutinin†	EU723762.1	18524884
Measles Fusion†	EU723763.1	18524884
Measles Matrix	NC_001498.1	19198563
Nipah Fusion†	AY816748.1	15972675
Nipah Glycoprotein†	AY816745.1	15972675

† indicates codon optimized sequence

Supplemental Table 3: NCBI sequences and references utilized for generation of adenovirus vectors. † indicates codon optimized sequence.

**Antibody**	**Catalog #**	**Assay**
MeV Matrix	Sigma-Aldrich; #MAB8910	IF, WB
MeV Fusion	Bioss; #BS-0886R	IF
MeV Nucleoprotein	Sigma-Aldrich; #MAB8906	IF, WB
dsRNA	Sciscons J2; #10010200	IF
ZO-1	Thermo Fisher Scientific; #33-9100	IF
E-cadherin	Cell Signaling Technology; #3195	IF
Vinculin	Thermo Fisher Scientific; #700062	WB
NiV Glycoprotein	Abcam; #ab322205	WB
SiR-Actin 647	Spirochrome; #CY-SC001	IF
Phalloidin-Alexa 647	Thermo Fisher Scientific; #A22287	IF

Supplemental Table 4: Primary antibodies utilized for immunostaining (IF) and/or immunoblotting (WB).
